# The fecal and oropharyngeal eukaryotic viromes of healthy infants during the first year of life are personal

**DOI:** 10.1038/s41598-022-26707-9

**Published:** 2023-01-17

**Authors:** Xaira Rivera-Gutiérrez, Patricia Morán, Blanca Taboada, Angélica Serrano-Vázquez, Pavel Isa, Liliana Rojas-Velázquez, Horacio Pérez-Juárez, Susana López, Javier Torres, Cecilia Ximénez, Carlos F. Arias

**Affiliations:** 1 Instituto de Biotecnologıía, Universidad Nacional Autónoma de México, Cuernavaca, Morelos Mexico; 2 Unidad de Investigación en Medicina Experimental, Facultad de Medicina, Universidad Nacional Autónoma de México, Mexico City, Mexico; 3 Unidad de Investigación Médica en Enfermedades Infecciosas y Parasitarias, Hospital de Pediatría, Centro Médico Nacional Siglo XXI, Instituto Mexicano del Seguro Social, Mexico City, Mexico

**Keywords:** Metagenomics, Viral infection

## Abstract

Using a metagenomic sequencing approach, we described and compared the diversity and dynamics of the oropharyngeal and fecal eukaryotic virome of nine asymptomatic children in a semi-rural community setting located in the State of Morelos, Mexico. Ninety oropharyngeal swabs and 97 fecal samples were collected starting 2 weeks after birth and monthly thereafter until 12 months of age. In both niches, more than 95% of the total sequence reads were represented by viruses that replicate either in humans or in plants. Regarding human viruses, three families were most abundant and frequent in the oropharynx: *Herpesviridae, Picornaviridae,* and *Reoviridae*; in fecal samples, four virus families predominated: *Caliciviridae, Picornaviridae, Reoviridae,* and *Anelloviridae.* Both niches showed a high abundance of plant viruses of the family *Virgaviridae*. Differences in the frequency and abundance of sequence reads and diversity of virus species were observed in both niches and throughout the year of study, with some viruses already present in the first months of life. Our results suggest that the children’s virome is dynamic and likely shaped by the environment, feeding, and age. Moreover, composition analysis suggests that the virome composition is mostly individual. Whether this constant exposition to different viruses has a long-term impact on children’s health or development remains to be studied.

## Introduction

With little more than 20 years of studies of the human gut microbiome, it is known that its bacterial component plays a crucial role in human health and early development, beginning colonization at early times after birth^1^. This colonization can be influenced by birth mode, and subsequently, by food ingestion, antibiotics use, lifestyle, age, and other factors^2^, and is highly dynamic up to the third year of life when it stabilizes to an adult-like configuration^3^.

On the other hand, the information about the viral component of the microbiome is more limited, particularly in children, on whom the studies have been mostly focused on the characterization of fecal bacterial viruses and their relationship with the bacterial communities^4^. This gap in knowledge increases when it comes to the eukaryotic virome, particularly of healthy infants. Transversal and longitudinal studies of virus diversity in fecal samples from newborns to 5-year-old children have been carried out using PCR amplification or metagenomics approaches, in general, it has been observed that the gut is rapidly colonized after birth by bacteria and their viruses, with eukaryotic viruses appearing 3–4 months later^2, 5–8^. Studies of infants in rural communities are even less common, and eukaryotic viruses have been described as sporadically present, with a tendency to increase or become more prevalent after the sixth month of age^9–12^. Other studies have focused on particular virus families and have reported the presence of plant viruses and caliciviruses in high frequency and abundance during the early life of children^13, 14^.

The virome composition in infants' body niches other than the gastrointestinal tract has not been extensively studied^15^. In particular, to our knowledge, no studies to date have assessed the oral eukaryotic virome of healthy children, and whether viruses are shared between the oropharynx and gut viromes is not known. The oropharynx is a hybrid space shared between the respiratory and digestive tracts, where air but also food and saliva pass through. Thus, it would be expected that at least some microorganisms would be shared between both tracts, as has been shown in previous bacteriome studies in adults^16, 17^.

Here, we characterized and compared the eukaryotic viral communities present in the oropharynx and feces of nine asymptomatic children during their first year of life, living in a semi-rural community. The diversity and dynamics of the eukaryotic virome in both niches were analyzed in samples collected monthly from each subject to establish a robust longitudinal profile of the changes that occur during this important phase of the infants' development.

## Results

### Cohort composition and samples analyzed

**Figure 1:**
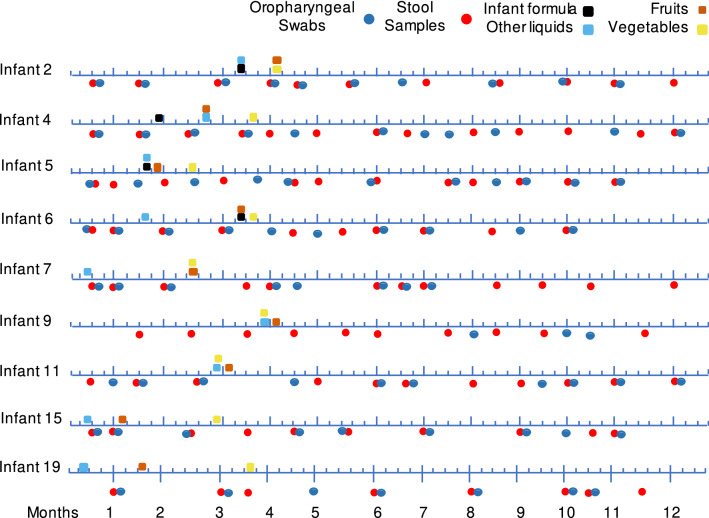
Timeline of the collection of stool samples (red circles) and oropharyngeal swabs (blue circles). The time is indicated as weeks and below is the equivalence in months. Squares illustrate the start of ingestion of food and liquids other than breastmilk.

This study was conducted in the village Xoxocotla, a semi-rural community in the State of Morelos, Mexico. The children were enrolled in our study between March 2015 and May 2017 and were followed during their first year of life. Fecal samples and oropharyngeal swabs were collected monthly, starting 2 weeks after birth and until 12 months of age. All the children were breastfed, and information about the introduction of other types of food (Fig. 1) and living conditions (Table S1) was recorded. Our cohort consisted of six females and three males, four were born via cesarean section, and five were delivered vaginally. None of the children received antibiotics during the study period, and they were vaccinated according to the Mexican vaccination program^10^. During the time of the study, no respiratory or gastrointestinal symptoms were reported for any of the infants in the cohort. In total, nucleic acids (both DNA and RNA) from 187 samples (90 oropharyngeal swabs and 97 fecal samples) were analyzed by next-generation sequencing. A large proportion of the viral sequence reads were assigned to prokaryotic viruses, with bacteriophages being 2:1 more abundant than eukaryotic viruses in the oropharynx and about 6:1 more abundant in fecal samples (Fig. S1). However, considering that the infants' eukaryotic virome is largely undescribed, this report is focused on the description of eukaryotic viruses, specifically on human and plant viruses which were the most frequent and abundant.

### The oropharynx eukaryotic virome

A total of 50 viral families were detected as part of the oropharyngeal virome (Fig. S2); only five of them represented more than 1% of the total viral sequence reads, and these five virus families accounted for 95% of the total species viral reads. Viruses in three of these families infect humans: *Herpesviridae*, *Picornaviridae,* and *Reoviridae*, while viruses in the *Virgaviridae* and *Totiviridae* families infect plants and fungi (Table S2). In general, of the total viral sequence reads at the family level, about 17% corresponded to human viruses and 80% represented viruses that infect mainly plants (Table S3).

**Figure 2:**
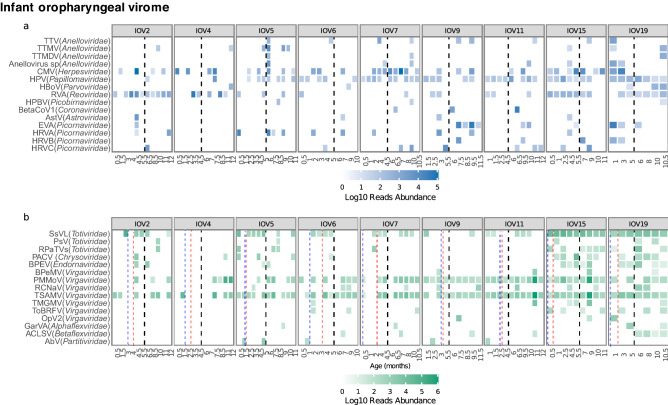
Infant oral virome (IOV). Normalized number of reads for viral species during the first year of life expressed in a logarithm base 10 scale. (**a**) Human viral species. (**b**) Plants and fungi viral species; species categorized as ‘sp.’ in Supplementary Table S3 were omitted from this figure. The family to which each species belong is shown in parenthesis. Each panel illustrates a child, with the identifier IOV at the top, and the age in months at the bottom. Black dotted lines divide the first and second halves of the first year of life. Blue dashed lines indicate the introduction of liquids other than milk, and red dotted lines the introduction of fruits, and in some cases vegetables. Species that infect humans: TTV, torque teno virus; TTMV, torque teno mini virus; TTMDV, torque teno midi virus; CMV, cytomegalovirus; HPV, human papillomavirus; HBoV, human bocavirus; RVA, rotavirus A; HPBV, human picobirnavirus; BetaCoV1, betacoronavirus 1; EVA, enterovirus A; Ast, astrovirus; HRVA, rhinovirus A; HRVB, rhinovirus B; HRVC, rhinovirus C. Species that infect plants: SsVL, *Scheffersomyces segobiensis* virus L; PsV, *Puccinia striiformis* totivirus; RPaTVs, red clover powdery mildew-associated totivirus; PACV, *Persea americana* chrysovirus; BPEV, bell pepper alphaendornavirus; BPeMV, bell pepper mottle virus; PMMoV, pepper mild mottle virus; RCNaV, rattail cactus necrosis-associated virus; TSAMV, tropical soda apple mosaic virus; TMGMV, tobacco mild green mosaic virus; ToBRFV, tomato brown rugose fruit virus; OpV2, opuntia virus 2; GarVA, garlic virus; ACLSV, apple chlorotic leaf spot virus; AbV, *Agaricus bisporus* virus.

Fifty different virus species were identified in the oropharynx, with each sample having an average and an s.d. of 8 ± 5 virus species and a median of 5563 (187 to 1,980,824) reads. Out of these 50 virus species, 15 were known to replicate in humans and 27 in plants. Regarding frequency, seven human species and 14 plant species were found in low frequency, being present in less than 10% of the samples (Table S2). On the other hand, seven species were highly abundant and/or frequent in the oropharynx (Fig. 2a): (1) betaherpesvirus 5 (cytomegalovirus) was detected in all children, in some cases during the early months of life (children 2, 4, 15, and 19), while in other children (5, 7, and 15), it was found in samples collected throughout the year of study. (2) Rotavirus A was found in almost all samples collected from children 2, 15, and 19; of interest, one sample of child 2, collected in the fourth month, was found to contain several genes compatible with RotaTeq vaccine viruses (Table S4). Enterovirus A was identified in five children; in child 9, it was detected in four consecutive months, suggesting a chronic infection, while in child 19 was found abundantly in the first month of life. An increase in their frequency through each quarter of life (8%, 9.5%, 11%, and 17%, respectively) was observed. (3) Rhinovirus A was found in six children, being present in one to four positive samples of each child. Human rhinovirus C was also frequently found, and human rhinovirus B was also detected but with a lower frequency. (4) Papillomavirus represents an interesting case since it was highly frequent, albeit at low abundance, when compared with other viruses; thus, it was detected in more than 80% of the samples of four children (7, 11, 15, and 19), including those collected during the first months of life (Fig. 2a, Table S2). Moreover, a decreasing tendency in its frequency was observed over time, being commonly found during the first trimester of life (68% of samples) and less frequently later: 62% in the second, 50% in the third, and 47% in the fourth trimester. The other three highly abundant and/or frequent virus species detected infect plants: pepper mild mottle virus, tropical soda apple mosaic virus, and a non-described virus, which by protein identity is closely related to the *Scheffersomyces segobiensis* virus, whose host are fungi (Fig. 2b, Table S2).

**Figure 3:**
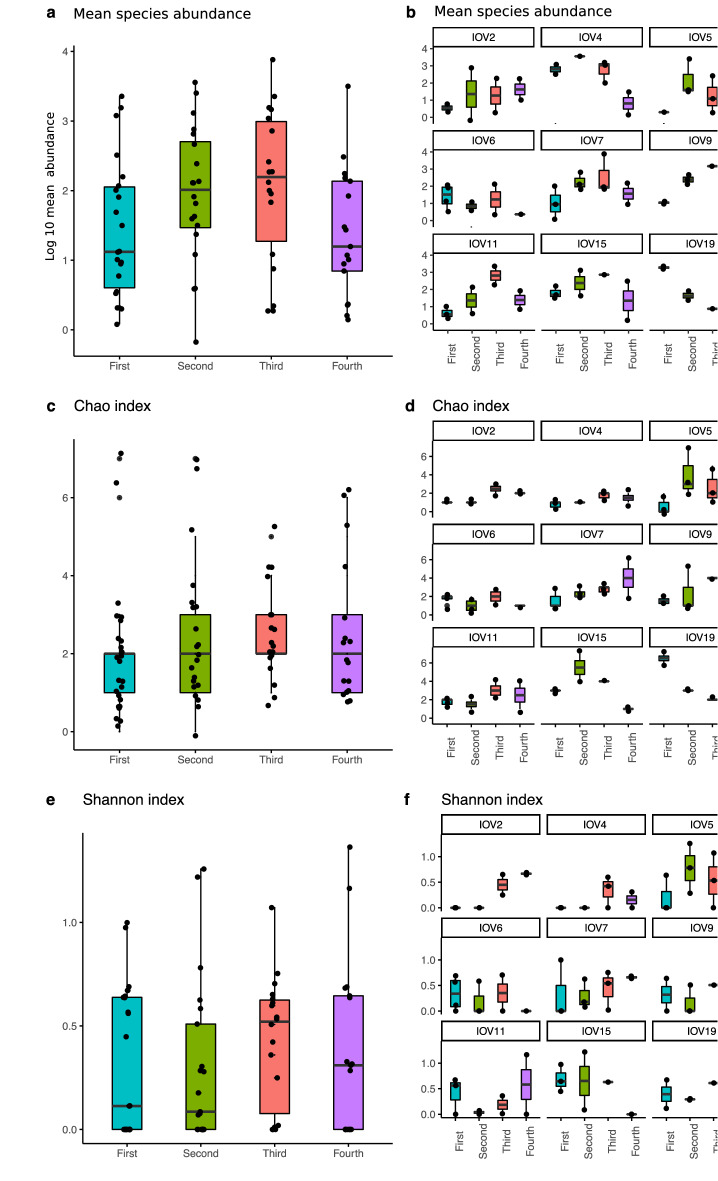
Bar plots comparing human eukaryotic viral diversity measures in the oropharynx analyzed by trimester of life, each point indicates a sample. (**a**) Mean of species abundance in all children samples, expressed in logarithm base 10; (**b**) Mean of species abundance per child, expressed in logarithm base 10; (**c**) Number of species in all children samples, calculated by the Chao richness index; (**d**) Chao richness index in each child; (**e**) Virus diversity in all children samples, calculated with the Shannon diversity index; (**f**) Shannon index of samples per child. In graphs (**b**), (**d**), and (**f**) the identifier IOV (infant oral virome) is at the top, and each quarter is indicated at the bottom.

Analysis of the means of human virus abundance between infants’ trimesters of life and within infants’ trimesters suggests an increase from the first trimester throughout the third; however, it was not statistically significant (Fig. 3a). Further, a per-child visualization showed that the distribution of viral abundance by trimester differs in each child, with some peaks in mean abundance that seem to be independent of age (Fig. 3b). When analyzing Chao richness (Fig. 3c,d) and Shannon diversity (Fig. 3e,f), no statistical significance was encountered across trimesters. Visualization and statistical analyses were also carried out considering birth mode, food intake, and living conditions, with no tendencies observed or significant results, suggesting that each child´s virome has an individual dynamic. Analysis of viral communities’ composition, using the Bray–Curtis distances matrix, reinforced this suggestion, with significant results obtained only when infants were compared along the year (PERMANOVA, R^2^ = 21.7%, p = 0.001).

### The fecal eukaryotic virome

Eukaryotic viruses from 46 different families were found in the fecal samples of the nine children (Fig. S3). However, 95% of the total viral sequence reads were represented by viruses belonging to only six families. Of these, four families infect humans: *Caliciviridae*, *Picornaviridae*, *Reoviridae,* and *Anelloviridae*, while the other two infect plants: *Virgaviridae* and *Tombusviridae* (Fig. S3, Table S5). In contrast with the oropharynx, human viruses represented a more significant proportion of the total viral sequence reads detected, with 46% of the reads at the family level (Table S3) and 68% at the virus species level (Table S5). Taxonomically, 46 different species of eukaryotic viruses were identified, with an average and an s.d. of 11 ± 5 species, and a median abundance of 5877 (121-5,288,674) sequence reads per sample. Of these species, 17 were identified as viruses that replicate in humans and 18 in plants. Overall, human and plant viruses were frequently observed in the samples, with the exception of 10 species that had less than 10% of frequency (Table S5). Ten of the 46 virus species represented 94% of the total reads; eight were of human origin and three were plant viruses (Table S5).

**Figure 4:**
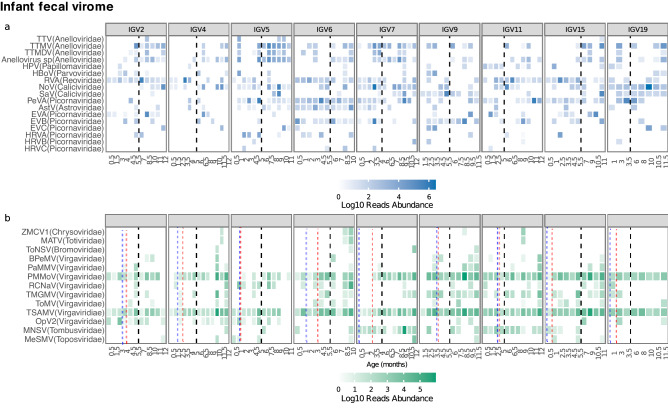
Infant gut virome (IGV). Normalized number of reads for viral species during the first year of life expressed in a logarithm base 10 scale. (**a**) Human viral species. (**b**) Plants and fungi viral species; species categorized as ‘sp.’ in Supplementary Table S6 were omitted from this figure. The family to which each species belong is shown in parenthesis. Each panel illustrates a child, with the identifier IGV at the top, and the age in months at the bottom. Black dotted lines divide the first and second halves of the first year of life, blue dashed lines indicate the introduction of liquids other than milk, and red dotted lines the introduction of fruits, and in some cases vegetables. Species that infect humans: TTV, torque teno virus; TTMV, torque teno mini virus; TTMDV, torque teno midi virus; HPV, human papilloma virus 1; HBoV, human bocavirus; RVA, rotavirus A; NoV, norovirus; SaV, sapovirus; PeVA, parechovirus A; AstV, mamastrovirus; EVA, enterovirus A; EVB, enterovirus B; EVC, enterovirus C; HRVA, rhinovirus A; HRVB, rhinovirus B; HRVC, rhinovirus C. Species that infect plants: ZMCV1, *Zea mays* chrysovirus; MATV, maize associated totivirus; ToNSV, tomato necrotic spot virus; BPeMV, bell pepper mottle virus; PaMMV, paprika mild mottle virus; PMMoV, pepper mild mottle virus; RCNaV, rattail cactus necrosis-associated virus; TMGMV, tobacco mild green mosaic virus; ToMV, tomato mosaic virus; TSAMV, tropical soda apple mosaic virus; OpV2, opuntia virus 2; MNSV, melon necrotic spot virus; MeSMV, melon severe mosaic toposvirus.

Regarding human viruses, noroviruses and sapoviruses, both in the *Caliciviridae* family, and a major cause of gastroenteritis in children under 5 years of age, were frequently found, being present in 57 (59%) and 28 (29%) of the samples, respectively (Table S5). Interestingly, both viruses were less prevalent in the first trimester of the year (45% for norovirus and 11% for sapovirus), increasing according to age until reaching a maximum in the fourth trimester, with 72% and 45% of prevalence, respectively. Noroviruses were also the most abundant species in the fecal samples, representing 32% of the total reads, while sapoviruses showed a 3.8% abundance (Fig. 4a, Table S5); a more detailed description of these species had been previously described^14^. Viruses from the *Picornaviridae* family were also detected: Parechovirus A was found in more than 75% of the samples of infants 5, 6, 7, and 15, while enterovirus A and enterovirus B were also frequently detected; in two children (6 and 9), enterovirus B was found nearly all along the year. Enterovirus C was found at low frequency, and viruses in six samples from five different children could be identified as poliovirus (children 2, 4, 5, 9, and 19; Fig. S4). Human rhinovirus A, B, and C was also detected in some samples with varying abundance.

In the *Reoviridae* family, Rotavirus A was frequently and abundantly identified, representing 9% of the total sequence reads (Table S5), with a prevalence of 67%; most samples from children 2, 6, 7, 11, and 15 were positive for the virus (Fig. 4a). Interestingly, a decrease in its prevalence was observed from the first through the third trimester of life, being 81%, 70%, and 42% prevalent, respectively. The high frequency during the first and second trimesters of life could be related to vaccination. Of note, rotavirus genes from bovine origin plus either VP4 and/or VP7 of human origin could be assembled and annotated in two samples of one child (15) and in one sample of children 2, 4, 5, 6, and 11 (Table S4), strongly suggesting that these viruses were derived from the RotaTeq vaccine.

Finally, within the *Anelloviridae* family, two species had a high frequency (49% and 50%), with low abundance (mean of 3.7% and 1.2%): torque teno mini virus and Anelloviridae sp. Both species were present in all children (Fig. 4a) and increased their frequency with age. These viruses were 26%, 44%, 61% and 72% for torque teno mini virus, and 11%, 40%, 85% and 77% for Anelloviridae sp during the initial four trimesters of life, respectively.

Regarding plant viruses, three species of viruses were also frequent and abundant: tropical soda apple mosaic virus, pepper mild mottle virus, and melon necrotic spot virus. (Fig. 4b and Table S5). A detailed description of the prevalence of plant viruses from the *Virgaviridae* family in a subset of the children included in this study has been previously reported^13^.

**Figure 5:**
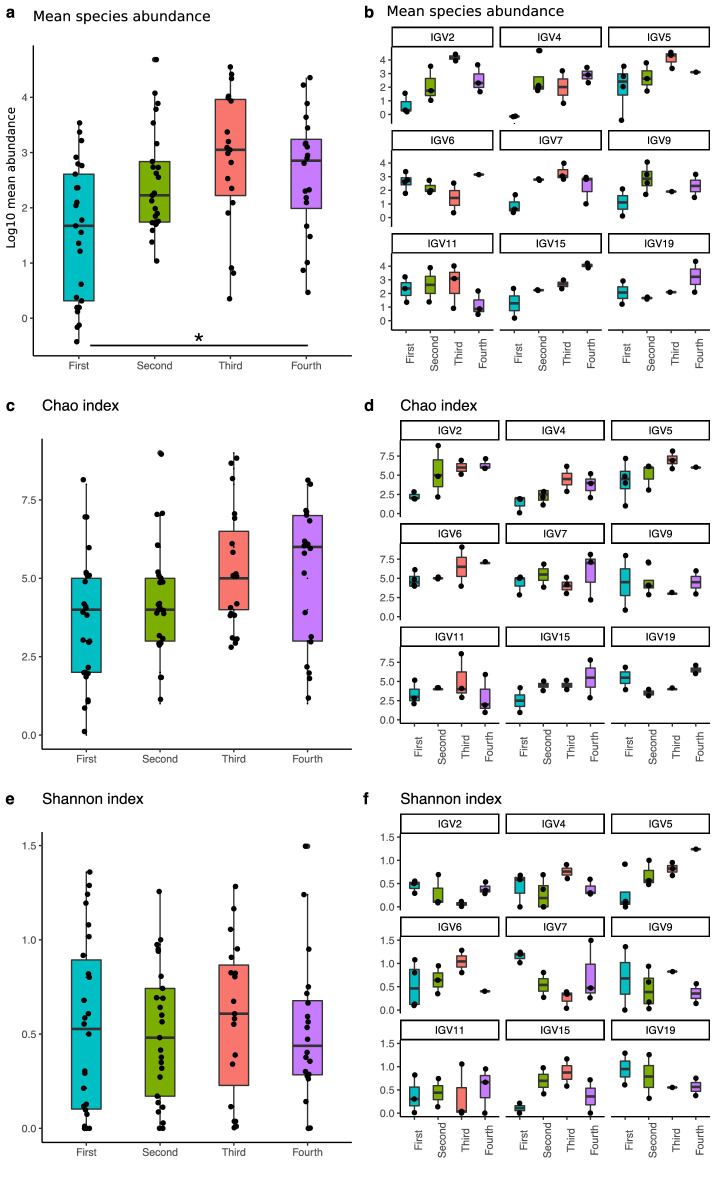
Bar plots comparing human eukaryotic viral diversity measures in fecal samples analyzed by trimester of life, each point indicates a sample. (**a**) Mean of species abundance in all children samples, expressed in logarithm base 10; *Represent p < 0.005 for paired comparisons; (**b**) Mean of species abundance per child, expressed in logarithm base 10; (**c**) Number of species in all children samples, calculated by the Chao richness index; (**d**) Chao richness index in each child; (**e**) Virus diversity in all children samples, calculated with the Shannon diversity index; (**f**) Shannon index of samples per child. In graphs (**b**), (**d**), and (**f**) the identifier IGV (infant gastric virome) is at the top, and each quarter is indicated at the bottom.

Alpha diversity analysis of the mean of virus abundances and Shannon diversity index, among and within infants’ trimesters, showed no statistical significance (Fig. 5a,e). A per-child visualization suggested that in six  children (2, 4, 5, 7, 15, and 19), there was an increase in abundance until the third trimester (Fig. 5b). However, a finer analysis showed a significant abundance increased only between and within the first and fourth infant trimester (permutational ANOVA for repeated measures p = 0.03 and p = 0.04, respectively). On the other hand, richness was different among children and also when the different trimesters of each child were compared (permutational ANOVA for repeated measures p = 0.01 and p = 0.0001, respectively; Fig. 5c,d). A graphical visualization suggests a global tendency of increase in richness by trimester of life (Fig. 5c). However, the individual visualization of richness suggests that changes in each infant are independent of age (Fig. 5d). No other statistical significance was found.

Regarding Beta diversity analysis, the viral community’s composition among trimesters showed significant differences (PERMANOVA R^2^ = 11.8%, p = 0.001). When a pairwise comparison was made, significant results were obtained between trimester first and second versus third and fourth (adjusted p = 0.0030 and p = 0.003 for the 1st and p = 0.013 and p = 0.012 for the 2nd trimester comparisons). Nevertheless, a higher variation was obtained when variation within the infants’ trimesters was evaluated (PERMANOVA R^2^ = 25.2%, p = 0.001).

### The oropharyngeal and fecal viromes have distinct virus composition and diversities

**Figure 6:**
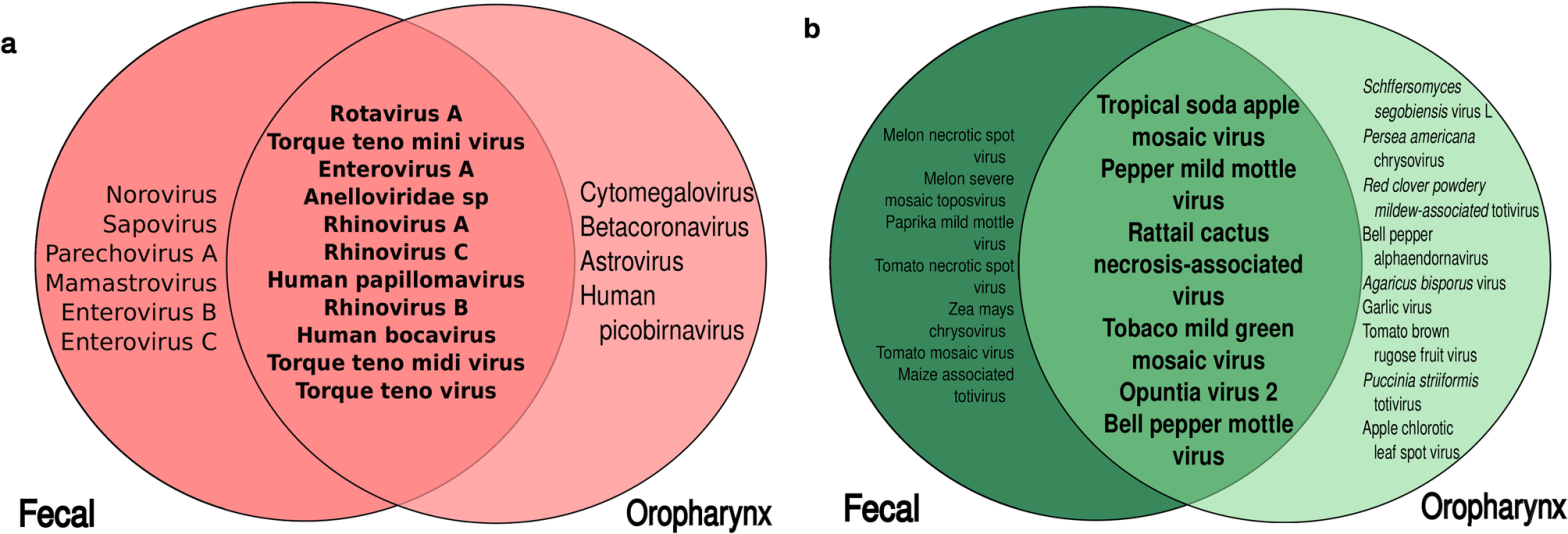
Venn diagram showing shared (in bold) and unique species in each tract that are known to infect humans (**a**) or plant and/or fungi (**b**). Species names are ordered according to their abundance, from highest to lowest in each area of the diagram.

When the host of origin of the viruses in each tract was analyzed, a clear difference was found. Sequence reads corresponding to human viruses predominated in feces, while those for plant viruses were dominant in the oropharynx (Table S3). Overall, ten species of human viruses were found in both tracts (Fig. 6a), and some showed differences in the abundance and timing of appearance in each tract. Rotavirus A was the most abundant of these shared viruses. Not surprisingly, the number of rotavirus reads was markedly different in both tracts, being 16 times higher in the feces as compared to the oropharynx (1,494,082 vs. 92,583 reads); accordingly, this virus was frequently found in fecal samples, while sporadically detected in the oropharynx, except in children 2, 15, and 19. Enterovirus A was present in both niches with low frequency and at timepoints not concurrent in the same child. In contrast, as expected for a respiratory virus, human rhinovirus A represented 7.3% of the human virus reads in the oropharynx compared to 0.3% in fecal samples (Tables S2 and S5). Torque teno mini virus and Anelloviridae sp were present in all children with a lower frequency and abundance in the oropharynx compared to feces. All other viruses detected in both tracts were found only sporadically.

Regarding viruses present uniquely in either tract, four species were particular to the oropharynx. Six were only detected in fecal samples (Fig. 6a). Some of these species stand out for having high frequency and abundance in the type of sample they were detected, such as norovirus in feces and cytomegalovirus in oropharyngeal samples, suggesting a clear compartmentalization.

For plant and fungi viruses, we observed a rich collection of virus species (26). Still, only five were shared between both tracts, all belonging to the *Virgaviridae* family (Fig. 6b). These include pepper mild mottle and tropical soda apple mosaic viruses, which were present in high frequency and abundance in both niches and all children, suggesting a continuous exposure of infants to these agents.

Oropharyngeal samples were found to have a lower amount of sequence reads than fecal samples (permutational ANOVA for repeated measures p = 0.001) statistically; this difference was also observed in each child (Fig. S5). Richness was different between niches, with the fecal samples having a higher number of viral species per sample (permutational ANOVA for repeated measures p = 0.001). This tendency was also observed in each child. Interestingly, the total number of different species was higher in the oropharynx (50 species vs. 46). Although it was not statistically different, in the visualization, the Shannon-index diversity seems to be slightly higher in fecal compared to oropharyngeal samples (Fig. S5).

The viral community’s composition between both tracts and within the infants' samples based on the Bray–Curtis distances matrix was significantly different (PERMANOVA R^2^ = 25.4%, p = 0.001 Fig. S6, and R^2^ = 12.8%, p = 0.001, respectively). This complex interaction between composition and diversity is derived, as shown above, from the individuality of each child. We suggest that although viral composition varies between niches, some children will have higher differences in composition and dynamics than others, as observed by the richness and diversity measures (Fig. S6), and per niche analysis (Figs. 3 and 5).

### Phylogenetic analysis shows a large diversity of viruses both in the oropharynx and the fecal viromes

To evaluate the genomic diversity of the different viruses circulating in the community, we carried out phylogenetic analyses and nucleotide pairwise sequence comparisons of those viruses that were more frequently found.

Four norovirus genotypes were detected in the community (GII.4, GII.7, GII.17, GI.3). Within a virus genotype, no large virus genome diversity was observed (less than 2% nucleotide difference among them). Of interest, all fecal samples from child 19 were positive for norovirus, with six included in the phylogenetic analysis; all of them resulted to be closely related within the GII.4 genotype, suggesting a chronic infection and prolonged shedding of the virus (Fig. S7). Similarly, two consecutive fecal samples from child 5 and two from child 7 showed closely related GI.3 sequences, also suggesting a single infection, and consistent with a previous report^14^. On the contrary, there are other cases, such as children 4 and 9, who in consecutive fecal samples had phylogenetically different viruses, suggesting multiple infections. Regarding sapoviruses, three different genotypes were identified. Interestingly, infant 15 had two different genotypes at the same time, suggesting a co-infection, while infant 5 had consecutive fecal samples that also corresponded to different genotypes, suggesting multiple infections. Finally, infants 7 and 9 were infected by the same virus at the same time (Fig. S8).

Figure S9 shows the genetic diversity of the VP4 and VP7 rotavirus genes in those samples where the G and/or P serotype could be assigned. The VP4 sequences could be clearly divided into three groups. Serotype P1A, probably associated with VP7 serotypes G1 to G4, which could be derived from the RotaTeq vaccine strains or from local human strains; serotype P1A, associated with VP7 serotype G12 from local strains, and; serotype P7, most likely of bovine origin, derived from the RotaTeq strains. On the other hand, VP7 sequences were grouped into four serotypes: G1, G3, or G4, from the vaccine or local strains, and serotype G6, most probably representing the vaccine VP7 gene from bovine origin. The limited number of samples with sufficient sequences assembled to be analyzed did not allow us to determine if the virus present in consecutive rotavirus-positive samples or in feces and oropharynx samples collected close in time are related to the same or independent virus exposures.

Astroviruses were not found as frequently as norovirus or rotavirus; however, all fecal samples of child 6 were positive for this virus. Phylogenetic analysis and pairwise comparison of astrovirus sequences showed that the viruses present in samples from months 2, 4.5, 5.5, and 6 of child 6 were all very similar (Fig. S10). This observation suggests a possible astrovirus chronic infection for about 6 months. The astrovirus identified in sample 10 of this same child was clearly different, belonging to the MLB clade.

Parechoviruses were frequently found in five children (5, 6, 7, 11, and 15), with at least 75% of their fecal samples being positive for this virus (Fig. 4a). However, viruses were quite genetically diverse both in samples from the same child as well as samples from different children. Only two similar parechovirus strains were detected, infecting two different children at a similar timepoint (5 and 6) (Fig. S11). In the case of enteroviruses, they were also very diverse; however, two virus-positive oropharyngeal samples of child 9 (7.5 and 9.5) had very closely related viruses, as well as two consecutive fecal samples (2.5 and 3.5) of the same child (Fig. S4). On the other hand, despite the high genetic diversity of anelloviruses in the fecal samples, three children (2, 5, and 9) had viruses with very similar genomic sequences in samples collected in consecutive times between January and March 2016, suggesting prolonged shedding of the virus (Fig. S12).

In the case of human papillomaviruses, although they were frequently found in the oropharynx of four children (7, 11, 15, and 19), only a few had enough reads to assemble contigs larger than 500 nucleotides or to reach coverages of more than 40% of the virus genome. Among the samples that met either of these criteria, a close genetic relationship between the virus strains detected in two oropharyngeal (6 and 9.5) and two fecal (0.5 and 1.5) samples of child 11 was noted (Fig. S13). These 4 samples were the only ones that could be classified for child 11, raising the possibility that the viruses in several other oropharyngeal and/or fecal samples of this same child could also be genetically related. Closely related viruses were also found in consecutive oropharyngeal samples (5 and 6) of child 19, and in three oropharyngeal samples (1, 3.5, and 9) of child 15. On the other hand, similar rhinovirus strains were detected neither in two consecutive oropharynx or fecal samples nor in samples taken from both tracts at similar timepoints (except for samples 2 and 3 from child 5, in which the virus seems to be the same), indicative of the large genomic diversity of these viruses (Fig. S14). The same applied to bocaviruses, although viruses in two consecutive samples of child 9 appear to be closely related (Fig. S15).

The phylogenetic trees and pairwise genomic comparisons for tropical soda apple mosaic virus and pepper mild mottle virus showed a high genetic diversity. Only in very few cases do there seem to be a similar virus strain in two consecutive samples from a given child. For instance, the tropical soda apple mosaic virus present in fecal samples from months 3.5 and 4 of child 7 appears to be the same (Figs. S16 and S17), suggesting a prolonged permanence of this virus strain in the gut or continued acquirement of the same or of a closely related virus from the environment. In the case of pepper mild mosaic virus, a high diversity was also found, although similar virus strains seem to be present in three consecutive samples (2.5, 3.5, and 4.5) of child 9, and in fecal samples 10.5 and 11, as well as in oropharyngeal sample 9 of child 15 (Figs. S18 and S19).

## Discussion

Most previous studies of the infant's fecal virome have focused on bacterial viruses; in general, these studies agree that the infant's gut is rapidly populated by phages/prophages that seem to be derived from the colonizing gut bacteria after the first weeks of life^6^. In this report, we center our analyses on the diversity and dynamics of eukaryotic viruses to help fill out the existing gap of information on the diversity and dynamics of these viruses in the gut and oropharynx of healthy infants during early development.

Regarding the oropharynx virome, we found a high number of virus species with low abundances, possibly reflecting in many cases viruses transiently present in the throat, because this is an anatomical space where air, saliva, and food are constantly passing through. In previous studies where the nasal, saliva, and periodontal plaque viromes were characterized, viruses from the *Anelloviridae, Papillomaviridae,* and *Herpesviridae* families were found frequently and abundantly^18–22^. It has also been reported that herpesvirus and papillomaviruses are responsible for the most common infections in the oral cavity of children^21^. In agreement with these reports, we observed that cytomegalovirus was detected frequently and in high abundance during the year of the study, even during the first months of life. In this regard, cytomegalovirus has been described to be present in breast milk, and mature milk (45–90 days postpartum) had an increased abundance (26%) of herpesviruses when compared to the 7–15 days postpartum milk (8.8%)^23^. Furthermore, newborn infection with cytomegalovirus has been pinpointed as a form of natural immunization, with rates ranging from 5.7 to 58% of mother–child transmission^24^. Also, interestingly, in a bioaerosol study in a daycare center, 7.8% of the air samples were positive for cytomegalovirus^25^. It is possible that these factors could contribute to the presence and abundance of viruses in the oropharynx of young children.

Due to its anatomical and physiological functions, the oropharynx is largely exposed to the environment, a factor relevant for determining the oral virome^20^. This probably explains the presence of virus species that were identified sporadically during this study, since these viruses most likely reached this niche by breathing or ingestion from different sources. This could also explain why no clear tendencies were observed regarding how the virome establishes besides the individuality of each child. An exception were human papillomaviruses, which were frequently found in the oropharynx of four children (7, 11, 15, 19) during the year of study.

Regarding the fecal virome of healthy children during their early life, metagenomic analysis of DNA viruses of a week-old child showed the presence of bacteriophages but not human eukaryotic viruses^6^. In the search for common gastrointestinal viruses by RT-qPCR in children in Gabon and the Republic of Ghana, who were followed from birth to 1 month of age^26^, no viruses were found in the meconium, but six of the fifteen infants harbored at least one enteric virus (rotavirus, enterovirus or adenovirus) in fecal samples collected up to 28 days after birth. In our study, the first sample, of six children (2, 4, 5, 6, 7, and 15), including one taken at 8 days of life, showed the presence of human and plant viruses (rotavirus, norovirus, enterovirus, pepper mild mottle virus and tropical soda apple mosaic virus), albeit at a low abundance.

In our cohort, human viruses in the *Caliciviridae*, *Picornaviridae*, *Reoviridae,* and *Anelloviridae* families were the most abundant. In other longitudinal studies in children younger than 1 year of age, similar results were reported^8, 9, 11, 12, 27–30^. Also, in a study of eight Belgian children picornaviruses, reoviruses, and anelloviruses were among the most abundantly detected^30^ and, in a cohort of 25 Australian children followed during their first 15 months of life^29^, 28% of the samples were RT-qPCR-positive for this virus, and its presence was associated with a low socioeconomic status. In our study, noroviruses were the most frequent and abundant intestinal viruses; although we did not classify our community based on income, the general population of Xoxocotla has a low socioeconomic level. A detailed description of our findings regarding caliciviruses has been previously published^14^. It is noteworthy that despite the frequent detection of caliciviruses and rotaviruses, none of the infants showed gastrointestinal symptoms during the study. As previously suggested^14^, sustained breastfeeding beyond 6 months of age could have helped with the low frequency of digestive and respiratory symptoms in these children, something we have found in previous studies in this community^31^.

Parechovirus A was detected in 59% of the samples of our cohort. This virus seems to circulate worldwide, as it was detected in approximately 2% of specimens from children in national enterovirus surveillance programs in Asia, Europe, and North America^32^, and was also described as highly prevalent (62%) in children under 1 year of age in Malawi^33^. Altogether, these results suggest that certain viruses can be found frequently in children, despite their health status, age, geographical region, and also despite the scope and methodology employed.

Rotavirus A has been described to be present either frequently^29, 34, 35^ or in a limited number of fecal samples^12, 26, 36, 37^ in several cohort studies of healthy children conducted in different countries. Of interest, in two of these studies the presence of vaccine strains was reported, derived from either the rotavirus monovalent Rotarix vaccine^27^ or the pentavalent RotaTeq^34^ vaccine. In our study, sequence reads related to bovine and human rotavirus genes, and/or with multiple G serotypes, were identified in 9 (14%) of the oropharyngeal or fecal samples positive for this virus, apparently related to virus components of RotaTeq, the vaccine that was administered in Xoxocotla during the time this study was carried out. It is noteworthy that seven of these samples were taken more than 18 days after the vaccine was administered (Table S4), suggesting a prolonged vaccine virus excretion in these cases. In the case of child 15 the rotavirus-positive fecal sample was detected 6 or 29 days after the second or third vaccine doses, respectively. Of interest, in both the oropharyngeal and fecal samples collected at 3.5 months of age for child 4, the vaccine virus was detected about 1 month after the first vaccine dose (Fig. S9 and Table S4). Whether this represents a prolonged shedding, or a transmission of the vaccine virus from another child recently vaccinated cannot be established. Sharing of rotavirus vaccine strains has been documented between brothers^38^, and shedding of RotaTeq vaccine viruses has been described, but not after 15 days post-vaccination^39^.

Of particular interest, two of the oropharyngeal samples (from children 2 and 4), which seem to contain RotaTeq vaccine viruses, were collected 19 and 28 days after their last vaccine doses, respectively (Table S5), raising the question of whether the rotavirus vaccine strains can replicate in the oropharynx; in this regard, rotavirus has been described in respiratory secretions in house transmission^40^ and in oropharyngeal aspirates of children hospitalized due to acute diarrheal rotavirus disease with respiratory symptoms^41^. Furthermore, it was recently reported that certain enteric viruses, among them rotavirus, can replicate in salivary glands and be excreted through saliva in a murine model^42^.

In agreement with studies that have identified viruses of the *Anelloviridae* family as the most frequent in fecal samples among children between 3–18 months of age^6, 7, 12^, we observed an escalated increase of torque teno mini virus and Anelloviridae sp each trimester, being the most frequent viral species among samples of the fourth trimester of life(Fig. 4, Table S6). The presence of anelloviruses has been associated with vaginal birth mode^9^, but no statistical differences between children born from vaginal or C-section were identified, although this might be due to our small sample size. We found torque teno mini virus, from the *Betatorquevirus* genus, to be among the most abundant in both tracts, although it was proportionally 13 times more abundant in fecal samples.

We have previously reported a high abundance and frequency of viruses from the *Virgaviridae* family in the oropharynx and gut in a subset of the children of our cohort^13^. Here, we extend these findings to our full cohort, confirming that children in the population, even at an early age, are continuously exposed to an extensive and highly diverse collection of plant viruses. Plant viruses were described to frequently circulate via bioaerosols in a daycare center, representing up to 94% of all the RNA viruses detected^25^. Although our children did not attend daycare facilities, they live in a semi-rural community where the main economic activity of the population is agriculture, which could explain the frequent and sometimes high amount of plant viruses present in the children's oropharynx and feces, although food ingestion, herbal medicines, and contaminated water could also be sources for these viruses^43^.

The composition of the virome was found to vary among samples but also among children, suggesting that each child has a unique and dynamic virome. There seems to be a slight tendency of increase in the abundance of viral reads and in the number of virus species in fecal samples after the first trimester of life, and probably also in oropharyngeal samples. The variation seems to have a multifactorial origin, but it is mainly shaped by the children's environment. These results are similar to those obtained when the fecal virome of twins was determined, showing that it is more similar between brothers when compared with non-related infants of the same age, suggesting that the virome is mainly shaped by the environment and living conditions^9, 44^.

Considering that several factors may contribute to the establishment of the fecal virome, the observation of tendencies that suggest a richer virus population after the first trimester makes sense. During the first 3 months of life the children have contact mainly with the mother or caretaker. Later, they start exploring their environment through their body, and weaning occurs. The observation that an increase in the abundance and frequency of viruses occurs after the first semester of life has been previously described^9, 11, 12^, however, in those studies the description has focused mainly on anelloviruses.

One of the salient features of our study was that all nine children shed a wide range of human enteric viruses asymptomatically during their first year of life, having up to 10 different viruses in a given sample. Also, some viruses could be detected in 80% or more of the total samples collected for a particular infant, including anelloviruses (TTMV and *Anellovirus* sp.), rotavirus A, norovirus, parechovirus A, astrovirus, and enterovirus B in the gut (Fig. 4), and human papillomavirus in the oropharynx (Fig. 2). This indicates that enteric viruses, as well as some respiratory viruses, can frequently be present for a long period of time in healthy children. Whether this continued detection of a particular virus in an infant represents prolonged shedding, is the result of a chronic infection, or is the consequence of repeated exposures to the virus has to be further explored. The constant shedding of diverse enteric viruses by healthy infants has been previously reported^26, 28, 35, 36, 45^.

The limitations of this study include treating fecal samples with 10% chloroform to remove bacterial cell contamination during nucleic acid isolation. This might represent a bias in our conclusions since lipid-containing viruses, although not very prevalent in the gut, could have been lost during this process. On the other hand, oropharyngeal samples were not chloroform-treated, and cytomegalovirus, an enveloped virus, was frequently detected. Also, the exclusion of the characterization of the bacterial viruses present in our samples, common components of the human virome, represents a limitation of our analyses. Finally, a limitation of this study is the low number of children enrolled, making it difficult to draw possible associations and conclusions about how the virome establishes, and to define a potential core virome. Nonetheless, our study provides a detailed blueprint of the virome dynamics and diversity in our studied population. Our findings suggest that each child has a ‘unique fingerprint’ virome, and show that the gut and oropharynx have a set of distinct and specific species, with some being shared among tracts. These findings are relevant to deepen our knowledge about the interaction among different viruses and their host and to further understand the composition and dynamics of viruses in the oropharynx and gut, and their interrelationship.

Whether the asymptomatic continuous contact of children with a wide range of human and plant eukaryotic viruses during early life, in a period when physiological, immunological, and developmental changes take place, has a role in modifying these processes, remains to be determined.

## Materials and methods

### Subjects and collection of samples

This study was conducted in collaboration with the community health clinic at Xoxocotla. Healthy pregnant women in their third trimester of pregnancy were invited to participate in this study. Written informed consent was obtained from all participants and/or legal guardians for study participation**.** The assurance of withdrawal at any time from the study was explained to each participant. At the moment of childbirth, clinic staff was notified and a subsequent in-home visit within 2 weeks after birth was made to start the collection of monthly oropharyngeal swabs and fecal samples. Additionally, growth, weight, height, vaccination, feeding, antibiotic intake, and symptoms were recorded weekly during the first 3 months of life, every 15 days from the fourth to the seventh month, and monthly thereafter. If intestinal o respiratory symptoms were reported, either for the mother or the infant, a new set of samples was taken. To ensure proper collection of fecal samples, the clinic staff trained the mothers to collect them in sterile cups, and plastic diapers were used inverted to avoid absorption of samples^14^. Characteristics of the children and household information were also registered^14^ (Table S1). If by the time of the visit there was no fecal sample, the clinical staff made a second visit the next day to collect the sample. Oropharyngeal swabs were taken with dry sterile swabs (rayon-tipped, BD BBL Culture Swab) and placed in 1 mL of viral transport medium (Microtest M4-RT, Remel). Both samples were kept at − 20 °C for a week at the community clinic and then at − 70 °C at the Institute of Biotechnology, National University of Mexico (IBt-UNAM)^10^. A total of 20 children were recruited for this study between 2015 and 2017, however, only nine children who had at least 80% of the planned samples collected were included in the analysis^14^.

### Ethical considerations

All the study protocols were performed in accordance with the relevant guidelines and regulations. This study was approved by the Ministry of Health of the state of Morelos, the scientific and ethics committee of the Medical School of UNAM (Project #088/2014), and by the bioethics committee of IBt-UNAM (Project #261). Approval of the committees was based on the Mexican Official Norm (NOM-012-SSA3-2007) as well as on the declaration of Helsinki from the World Medical Association.

### Nucleic acid isolation and sequencing

Nucleic acids (DNA and RNA) were extracted from feces and oropharyngeal swabs. Sterile water was used as a negative control. Stool samples were homogenized using a bead beater (Biospec Products, USA) with a mixture of 100 mg of 150–212 μm glass beads (Sigma, USA) and 10% chloroform in 1 ml of phosphate-buffered saline (PBS); oropharyngeal samples were not treated with chloroform. Then, fecal, oropharyngeal, and control samples were centrifuged at 2000×*g* and the supernatant was filtered using Spin-X 0.45 μm pore filters (Costar, NY) centrifuging at 10,000×*g* for 10–15 min. The filtered samples were then treated with turbo DNAse (Ambion, USA) and RNAse (2U) (Sigma, USA) for 30 min at 37 °C. Nucleic acids in the samples were extracted using PureLink viral RNA/DNA extraction kit (Invitrogen, USA) and eluted in nuclease-free water. cDNA was obtained using SuperScript III reverse transcriptase (Invitrogen, USA) with primer 5′-GTTTCCCAGTAGGTCTCN9-3′, and the second strand was generated by two rounds of synthesis with Sequenase 2.0 (USB, USA), using the same primer. The obtained dsDNA was amplified by 10 cycles with primer 5′-GTTTCCCAGTAGGTCTC-3′, with Phusion High Fidelity Polymerase (Finnzymes). The amplified DNA and cDNA were cleaned with ZYMO DNA clean and concentration 5 kit. Sequencing libraries were fragmented at 200 pb using Nextera XT DNA (Illumina). Sequencing was performed in a NextSeq500 Illumina platform at the National Laboratory for Technological Support to Genomic Sciences-Conacyt-UNAM^10, 13, 14^, generating 75 bp pair-end reads and a mean depth of 29,015,329 and 20,464,999 reads for oropharyngeal and fecal samples, respectively^10, 13, 14^.

### Bioinformatic analysis

An in-house bioinformatic analysis was used^10^. Briefly, adapters, low-quality bases, low complexity reads, and reads shorter than 40 bp were removed using FastP^46^ v0.19.4, discarding 3% of reads of oropharyngeal and 6% for fecal samples. Then, as recommended^47^, PCR exact duplicated reads were removed using Cd-Hit-Dup^48^ v4.6.8, eliminating 75% and 76% of reads, respectively. Human genome reference (GRCh38.p13, Genbank)^49^ and Silva release 132 database^50^ were used to filter human host (removing 2.3% and 0.71%, respectively) and ribosomal reads (16% and 3%) using Bowtie2^51^ v2.3.4.3^50^. After this process, a mean of 1,204,867 ± 868,988 oropharyngeal and 4,049,622 ± 2,612,729 gastrointestinal filtered sequence reads per sample remained (Table S6).

In order to identify reads of viral origin, the filtered reads were mapped against a nucleotide viral database obtained from NCBI^52^, using BbMap^53^ v38.26, and then assembled into contigs using SPAdes^54^ v3.14.1. Then, contigs were clustered at 95% of identity using CD-HIT^48^ v4.6.8 to obtain unique sequences. For taxonomic classification, assembled contigs, with read magnitude, and single reads were aligned using BLASTn^55^ v2.11.0 against the complete nt database, with an E-value of 0.001, 80% of query coverage, 70% of identity, and 20 hits maximum for each read to reduce false positives. Finally, reads that did not have a hit were extracted, assembled into contigs of a minimum size of 200 nt (with read magnitude), ORF-predicted using Prodigal^56^ v2.6.3, and then aligned against all nr protein database, using Diamond^57^ v9.9.22 with the same coverage and number of hit as blast. All used reference databases were updated in March 2020. Finally, BLASTn and BLASTx results were imported to MEGAN^58^ v6.19.7, in its naive mode with the Last Common Ancestor Algorithm (LCA) parameters set to 10% best hits threshold and minimal support hit of 4 for a taxon. Further, random samples with a high abundance of reads assigned to a specific species were manually verified. Taxa were extracted from MEGAN into a raw count matrix. Rotavirus genomes presented in Table S4 were genotyped using the rotavirus A genotype determination web-based tool from the NIAID pathogen database and analysis resource (ViPR)^59^.

### Statistical analysis

To reduce differences due to uneven sequencing depth, reads were normalized to reads per million (RPM) at 5 million reads per sample^10^. Five percent of outliers samples, with the minimum and the maximum number of reads, were discarded for both niches, excluding samples from the first trimester of life, thus remaining 81 oropharyngeal and 90 fecal samples. Only viruses that are known to replicate in humans were considered for the analyses. All statistics were performed in R^60^ v3.6.3, using the Vegan package^61^, unless indicated otherwise. Plotting was made using ggplot^62^ and cowplot^63^ packages.

For alpha diversity, mean abundances, Chao1 richness, and Shannon diversity index were calculated. Variance comparisons of these metrics among trimesters of life in infants and between niches were calculated with the non-parametrical aovp function in the lmPerm package for R, with default permutations (5000)^64^; in comparisons within infants and between them, quarter or niche was set as a fixed factor, the infants and the time (months) as random factors, with time nested within infants to consider repeated measures. Feeding variables (infant formula, water, fruits, among others) and before-and-after time of alpha metrics, were compared using the Wilcoxon test for paired samples. Grouping of infants by categorical variables (birth mode, weight percentile, water consumption, among others) was contrasted with the U-Mann Whitney test using specific timepoints: the first sample taken, or samples corresponding to the 3rd, 6th, 9th, or 12th month.

For beta diversity analysis, Bray–Curtis index was calculated. Distances matrix was used to compare variables, using the nonparametric multivariate permutation test (PERMANOVA), with Adonis function (1000 strata permutations) and pos-hoc pair-wise Adonis, with false discovery rate adjusted p value. Variance distributions were verified with the betadisper function. PCoA analysis and centroids were calculated with the ordispider function. In all analysis, a p-value < 0.05 was considered statistically significant.

### Assembly of viral genomes and phylogenetic analysis

To analyze the genetic variation of the predominant viruses detected in this study, all viral species with at least 20% genome coverage in a sample were considered in the following analysis. First, the corresponding reference genome sequence was downloaded from GenBank. Next, reads assigned to the species in each sample were mapped using Bowtie2 v2.3.4.3 to obtain consensus genomes with the iVar program (v1.3.1)^65^, using Phred score Q > 20 and a minimum read coverage depth of 3 × to call a base, with the majority base rule or N for lower values. Then, sequences were aligned with the reference genome and an externally related sequence to root the tree, using MAFFTv7 (30) program, and 5′ and 3′ regions were removed manually from the alignment. Finally. the sequence alignment was then used to reconstruct a maximum likelihood phylogeny tree, with 1000 bootstraps, using iqtree v.2.1.1^66^, evaluating the evolution model that best fit each alignment. Only aligned consensus with more than 40% of genome coverage were considered as input for MEGAX^67^ nucleotide pairwise comparisons.

## Supplementary Information

Supplementary Tables.

Supplementary Figures.

## Data Availability

Sample raw sequences (SRA) are available at BioProject under accession number PRJNA592261 at NCBI under accession numbers: SRX15720109 to SRX15720167, SRX11104216, SRX11104217, SRX11104202 to SRX11104214, SRX11104188 to SRX11104200, SRX11104074 to SRX11104186, SRX11104160 to SRX11104172, SRX11104146 to SRX11104158, SRX11104141 to SRX11104144, SRX7350247 to SRX7350268, SRX7350231 to SRX7350242, SRX7283366 to SRX7283371 and SRX7283335 to SRX7283363.
