# Supplementary material for: The fecal and oropharyngeal eukaryotic viromes of healthy infants during the first year of life are personal
Source: Sci Rep. 2023 Jan 17;13:938. doi: 10.1038/s41598-022-26707-9 (PMC9845211; doi:10.1038/s41598-022-26707-9)
Supplement: Supplementary file 2 — Supplementary Figures. [file 41598_2022_26707_MOESM2_ESM.pdf]

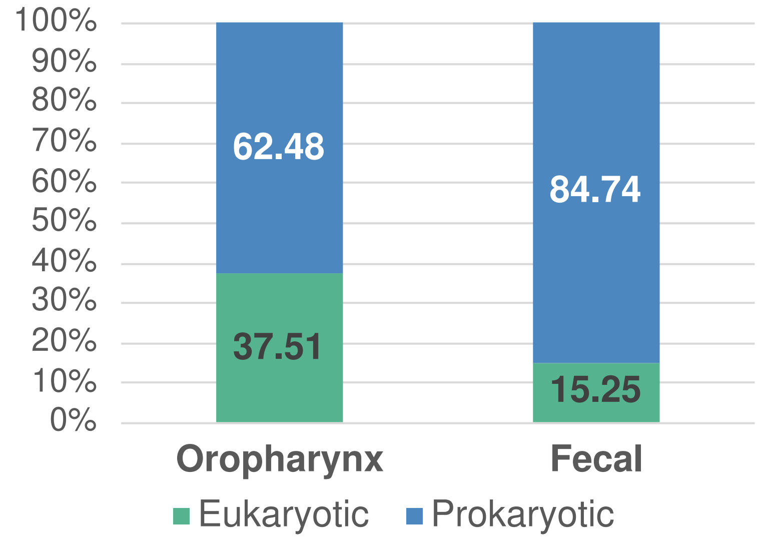


Fig. S1. Percentage of reads identified at the taxonomical level for eukaryotic and prokaryotic viruses in oropharyngeal and fecal samples.

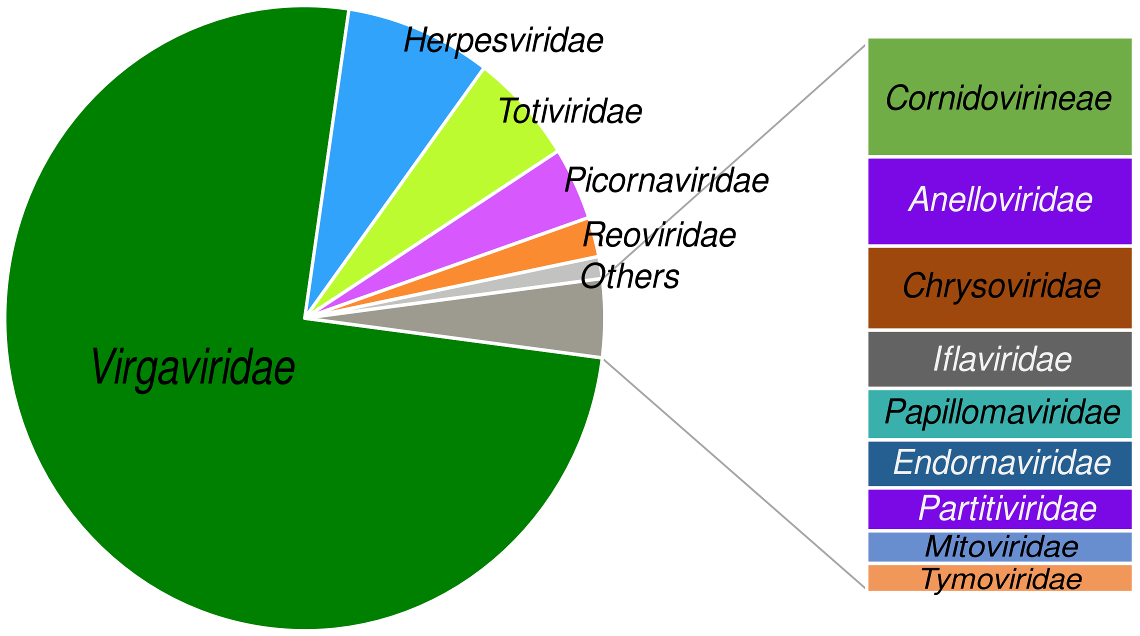


Fig. S2. Percentage of sequence reads in families, in which viral species were identified in the oropharyngeal samples. Families with less than 0.1 percentage of abundance were collapsed in "others".

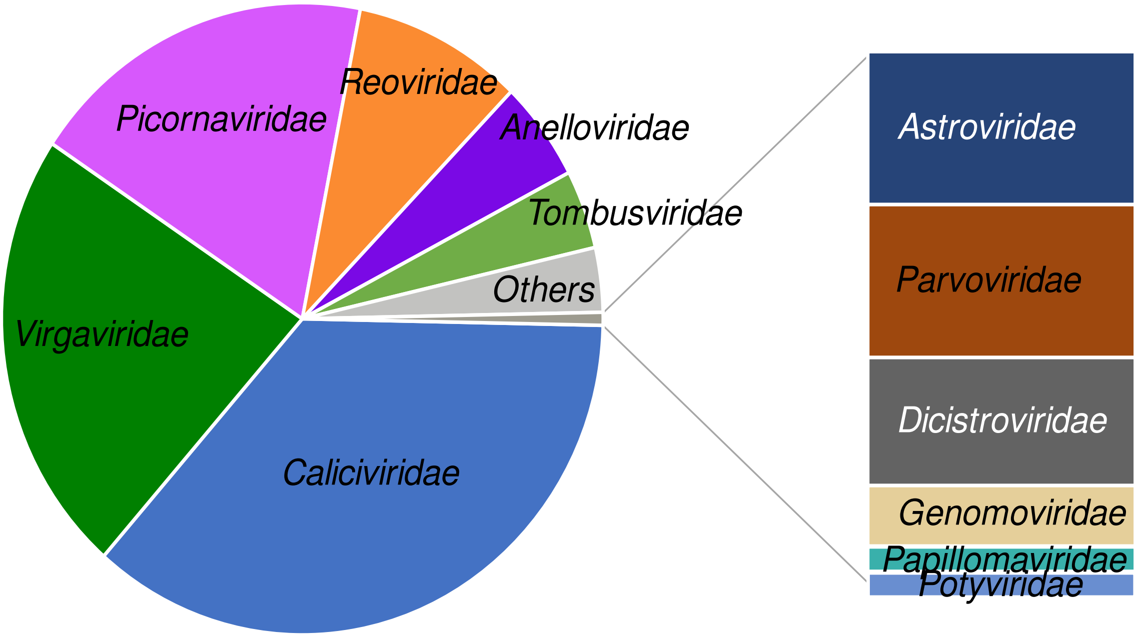


Fig. S3. Percentage of sequence reads in families, in which viral species were identified in fecal samples. Families with less than 0.1 percentage of abundance were collapsed in "others".

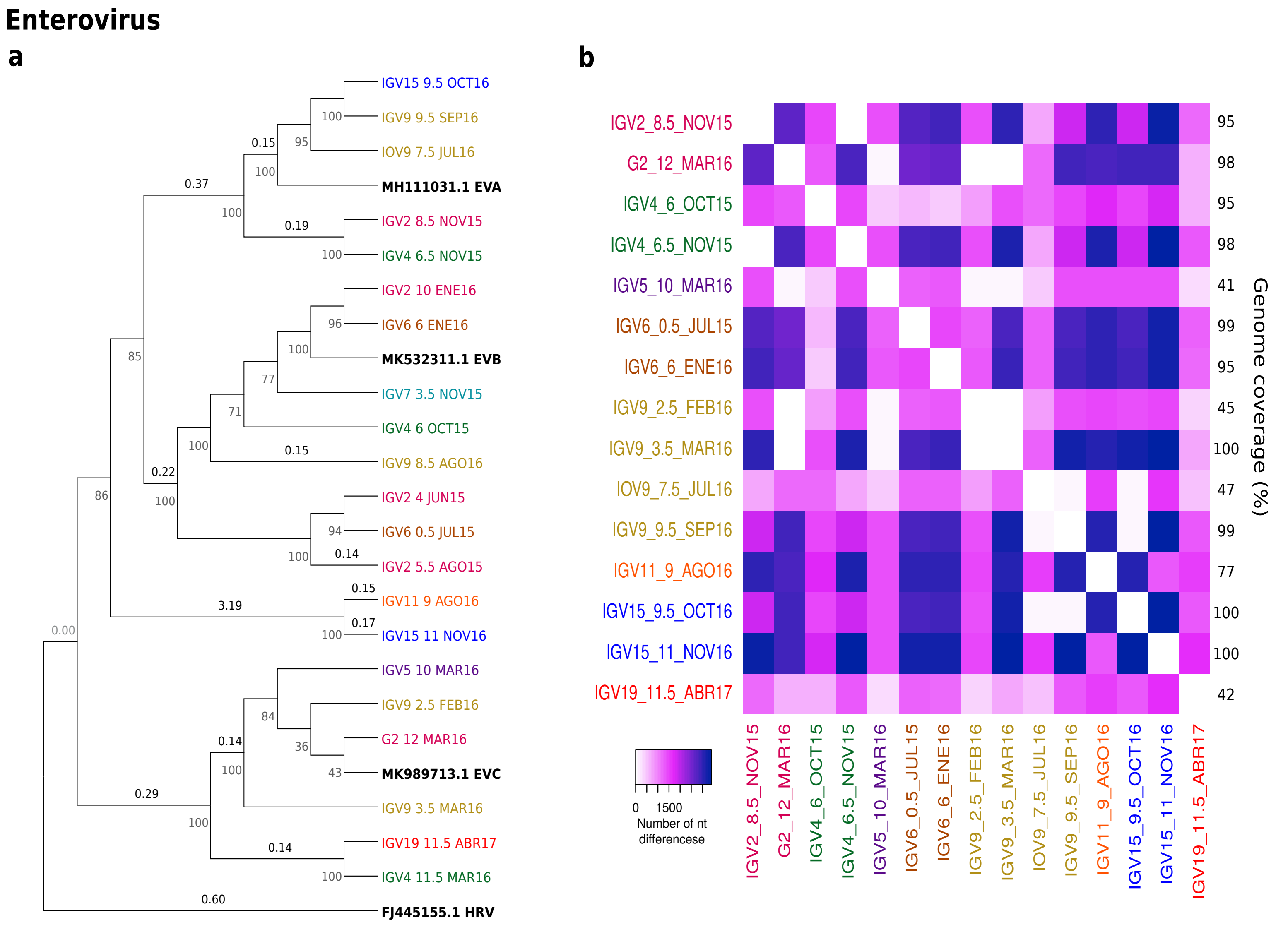


Fig. S4. Enterovirus genetic diversity. (a) Phylogenetic tree of complete genomes and partial sequences (contigs > 500 nt) constructed with GTR+F+R2 model. (b) Heatmap showing pairwise nucleotide differences of viral sequences with at least 40% of the genome coverage. Reference and rooted strains are in bold. Each children has an assigned color and names follow the next code: "IOV19_5 SEP16" indicates Infant Oropharynx Virus collected from child 19 at 5 months of age, on September 2016; "IGV11_1.5 DEC15" indicates Infant Gastrointestinal Virus collected from child 11 at 1.5 months of age, on December 2015.

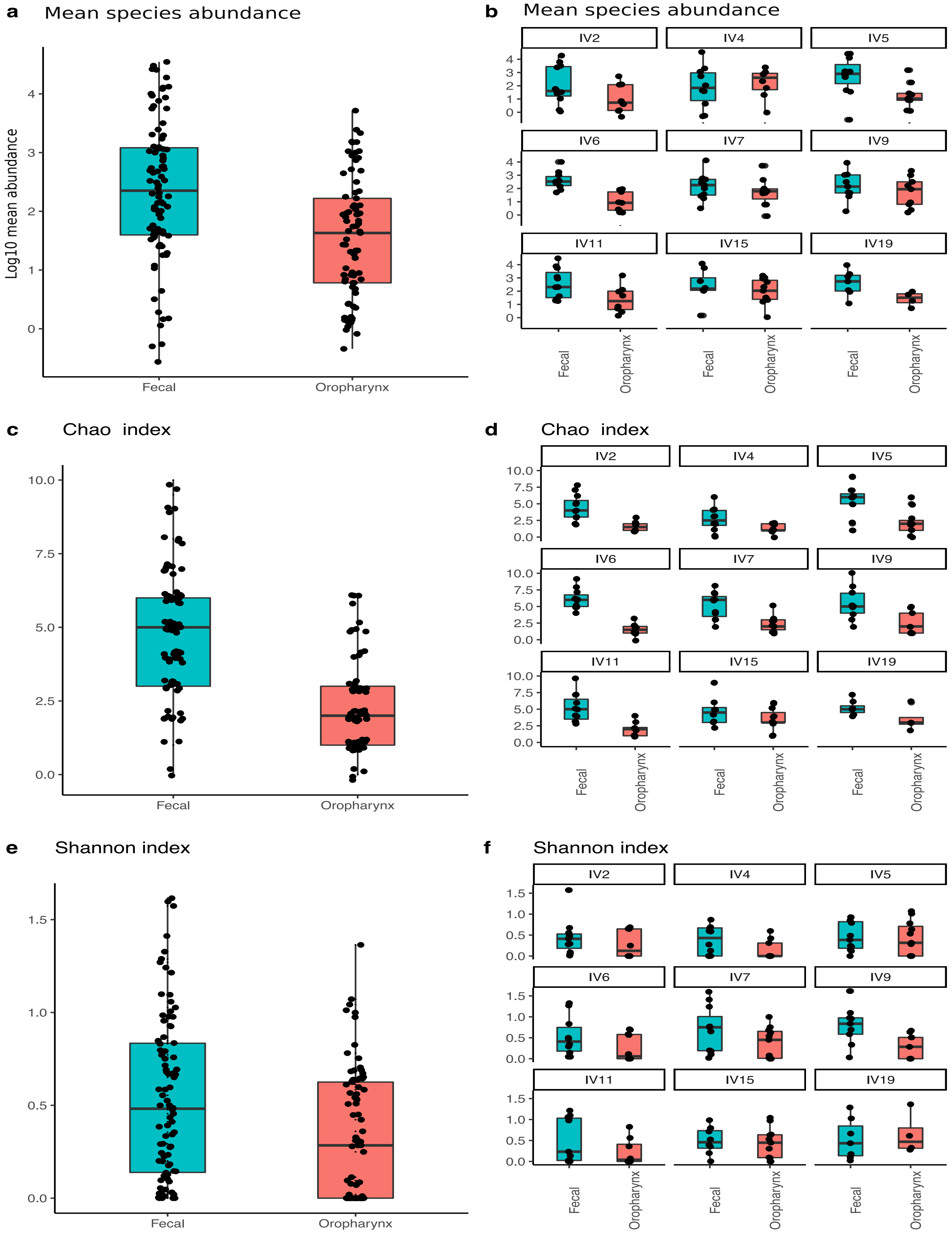


Fig. S5. Bar plots representing human eukaryotic viral diversity measures between oropharyngeal and fecal samples, each point represents a sample. (a) Mean of species abundance in all children samples expressed in logarithm base 10; (b) Mean of species abundance per child expressed in logarithm base 10; (c) Number of species (richness) in all children samples, calculated using Chao richness index; (d) Number of species in each child, calculated using Chao richness index; (e) Virus diversity in all children samples calculated with Shannon diversity index; (f) Shannon index of diversity of samples per child. In graphs b, d and f the identifier IV (infant virome) is at the top, and each quarter is indicated at the bottom.

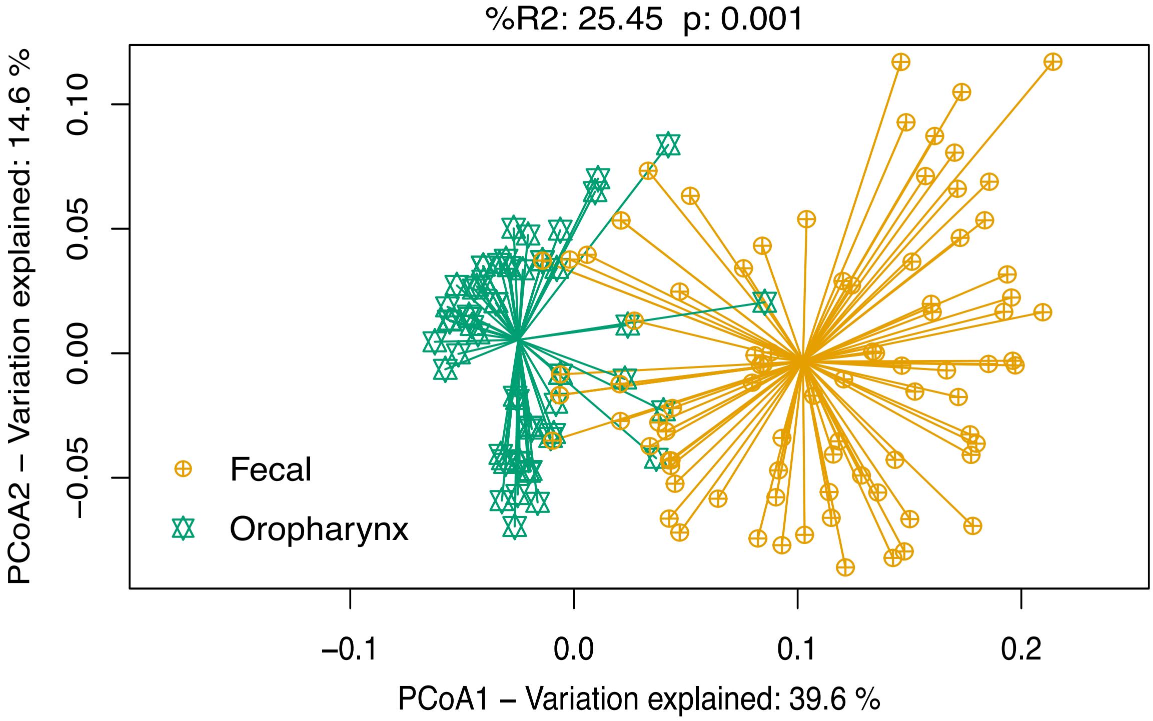


Fig. S6. Principal coordinate analysis (PCoA) based on normalized eukaryotic viral species counts per sample. Each point represents a sample and the colors represent either oropharyngeal or fecal samples. Centroid and dispersion of samples is showed for each type of sample (lines); PERMANOVA with 1000 bootstraps indicated that variation between tracts is 25.4%. Beta-diversities analyses are based on Bray-Curtis dissimilarities index for all samples.

# Norovirus

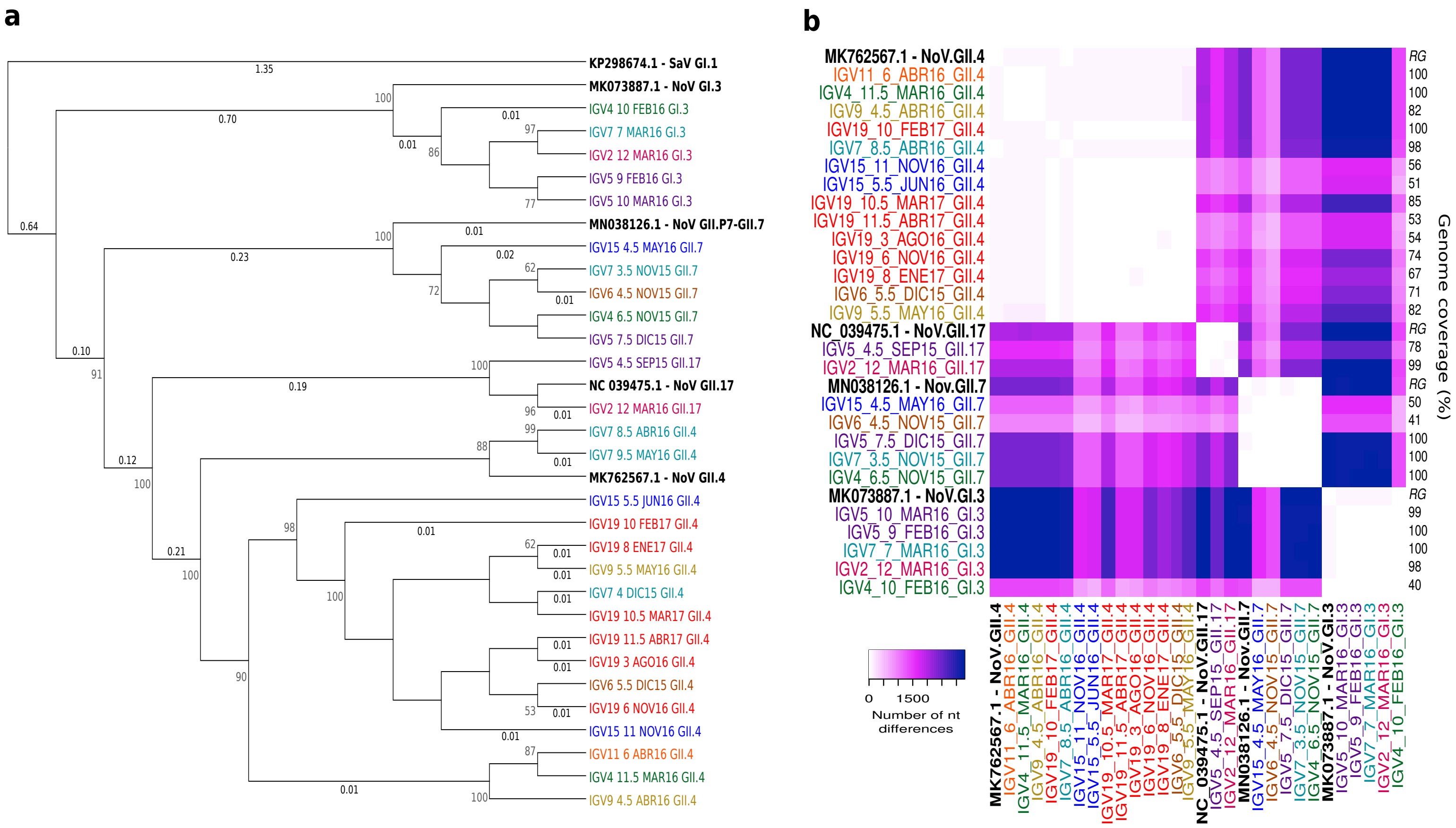


Fig. S7. Norovirus genetic diversity. (a) Phylogenetic tree of complete genomes and partial sequences (contigs > 500 nt) constructed with GTR+I model. (b) Heatmap showing pairwise nucleotide differences of viral sequences with at least 40% of the genome coverage. Reference and rooted strains are in bold. Each children has an assigned color and names follow the next code: "IOV19_5 SEP16" indicates Infant Oropharynx Virus collected from child 19 at 5 months of age, on September 2016; "IGV11_1.5 DEC15" indicates Infant Gastrointestinal Virus collected from child 11 at 1.5 months of age, on December 2015.

# Sapovirus

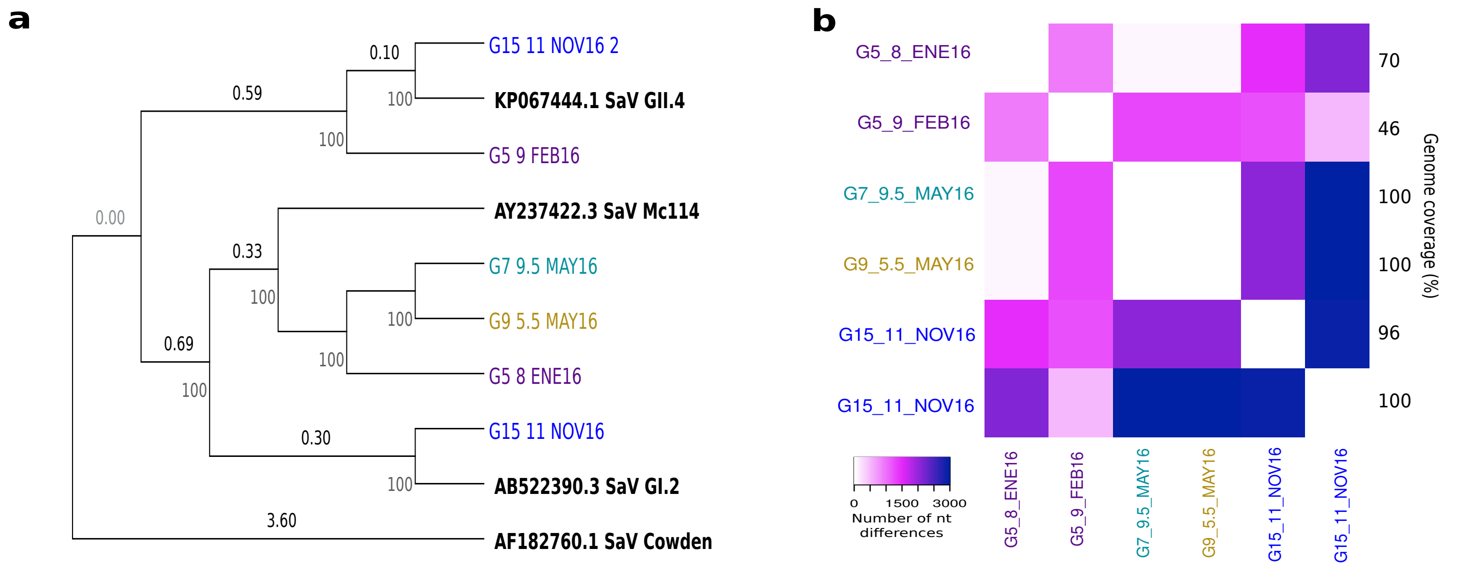


Fig. S8. Sapovirus genetic diversity. (a) Phylogenetic tree of complete genomes and partial sequences (contigs > 500 nt) constructed with TIM1+F+G4 model. (b) Heatmap showing pairwise nucleotide differences of viral sequences with at least 40% of the genome coverage. Reference and rooted strains are in bold. Each children has an assigned color and names follow the next code: "IOV19_5 SEP16" indicates Infant Oropharynx Virus collected from child 19 at 5 months of age, on September 2016; "IGV11_1.5 DEC15" indicates Infant Gastrointestinal Virus collected from child 11 at 1.5 months of age, on December 2015.

# Rotavirus VP4 Segment

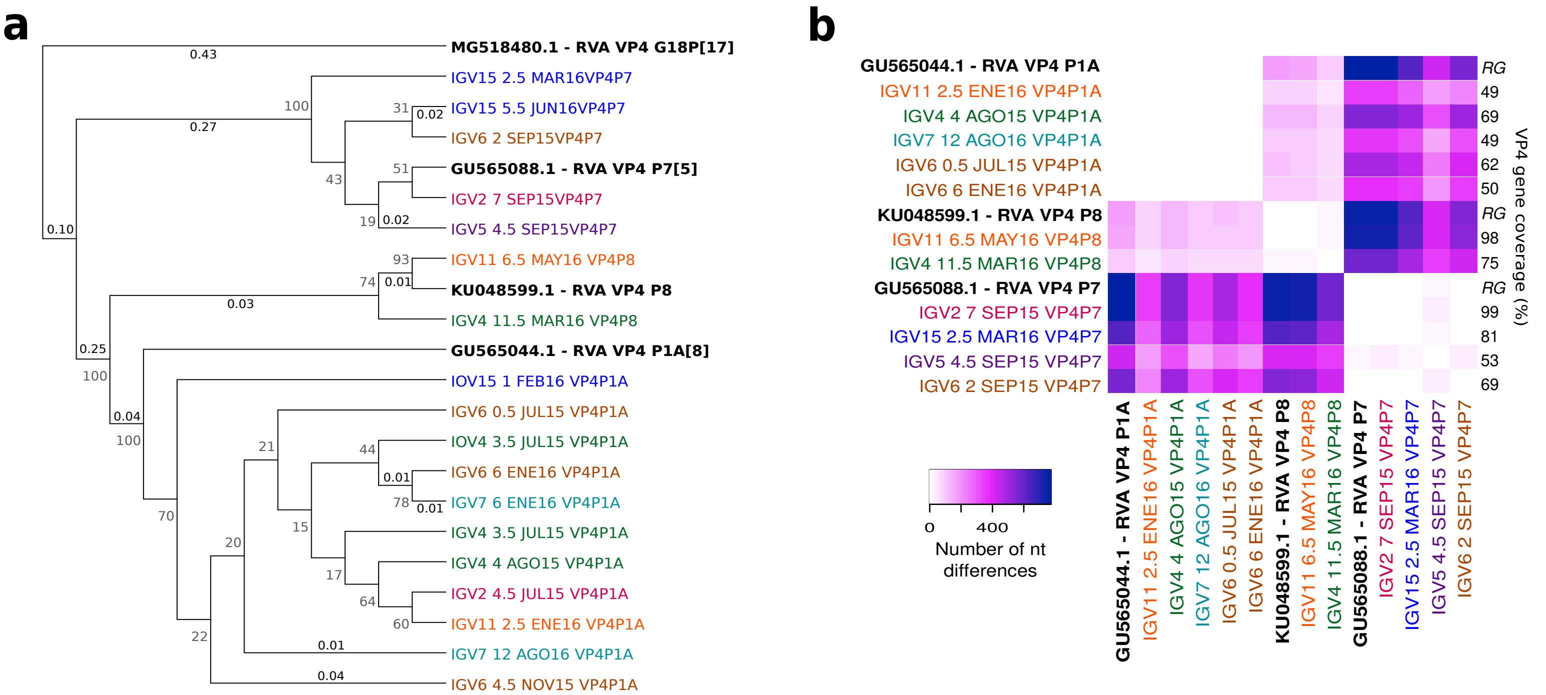


# Rotavirus VP7 Segment

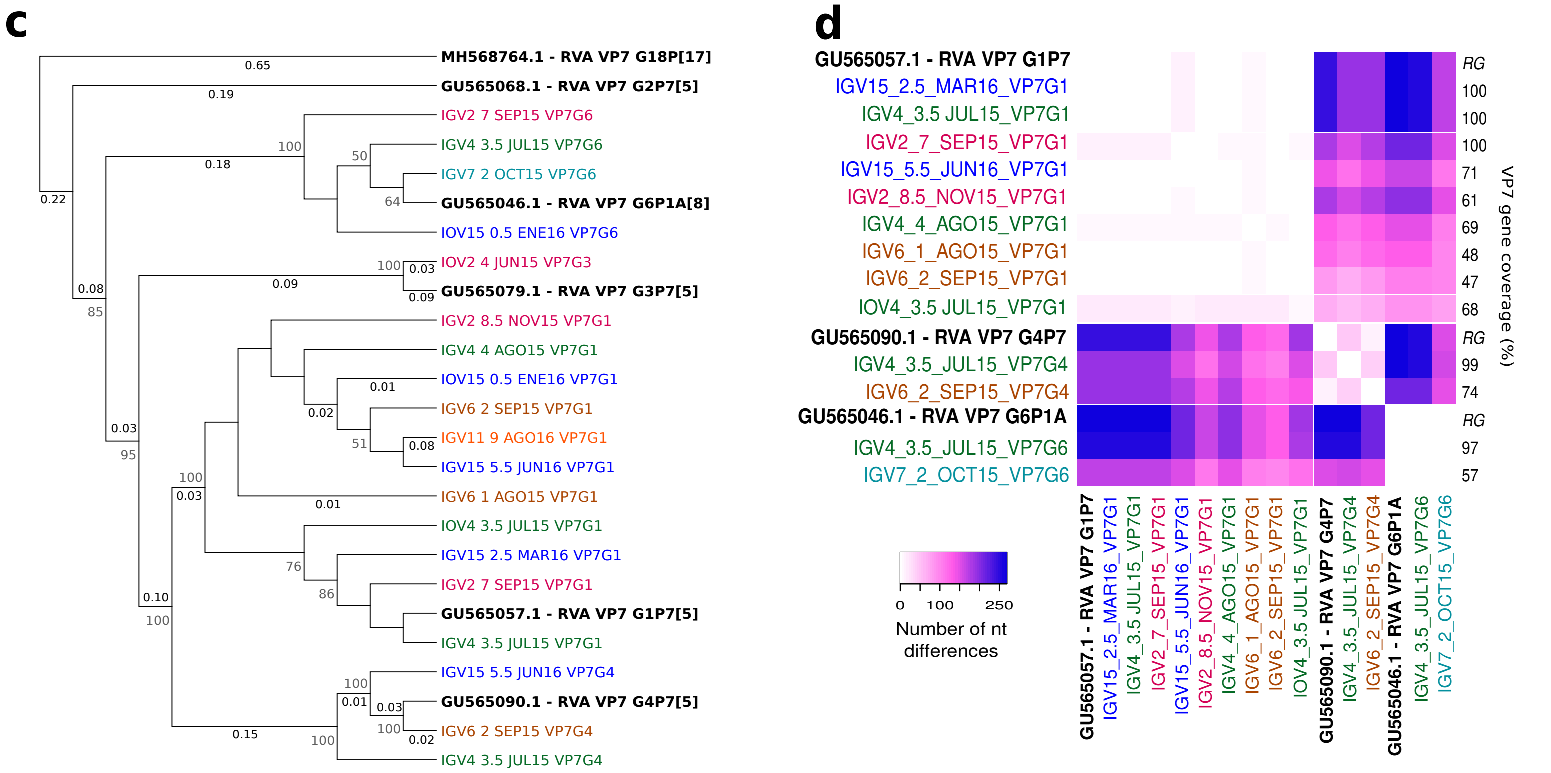


Fig. S9. Rotavirus VP4 and VP7 segments diversity. (a) Phylogenetic tree of VP4 complete gene and partial sequences (contigs > 500 nt) constructed with HKY model. (b) Heatmap showing pairwise nucleotide differences of VP4 gene sequences with at least 40% of the genome coverage. (c) Phylogenetic tree of VP7 complete gene and partial sequences (contigs > 500 nt) constructed with HKY + I model. (d) Heatmap showing pairwise nucleotide differences of VP7 gene sequences with at least 40% of the genome coverage. Reference and rooted strains are in bold. Each children has an assigned color and names follow the next code: "IOV19_5 SEP16" indicates Infant Oropharynx Virus collected from child 19 at 5 months of age, on September 2016; "IGV11_1.5 DEC15" indicates Infant Gastrointestinal Virus collected from child 11 at 1.5 months of age, on December 2015

# Astrovirus

**a**

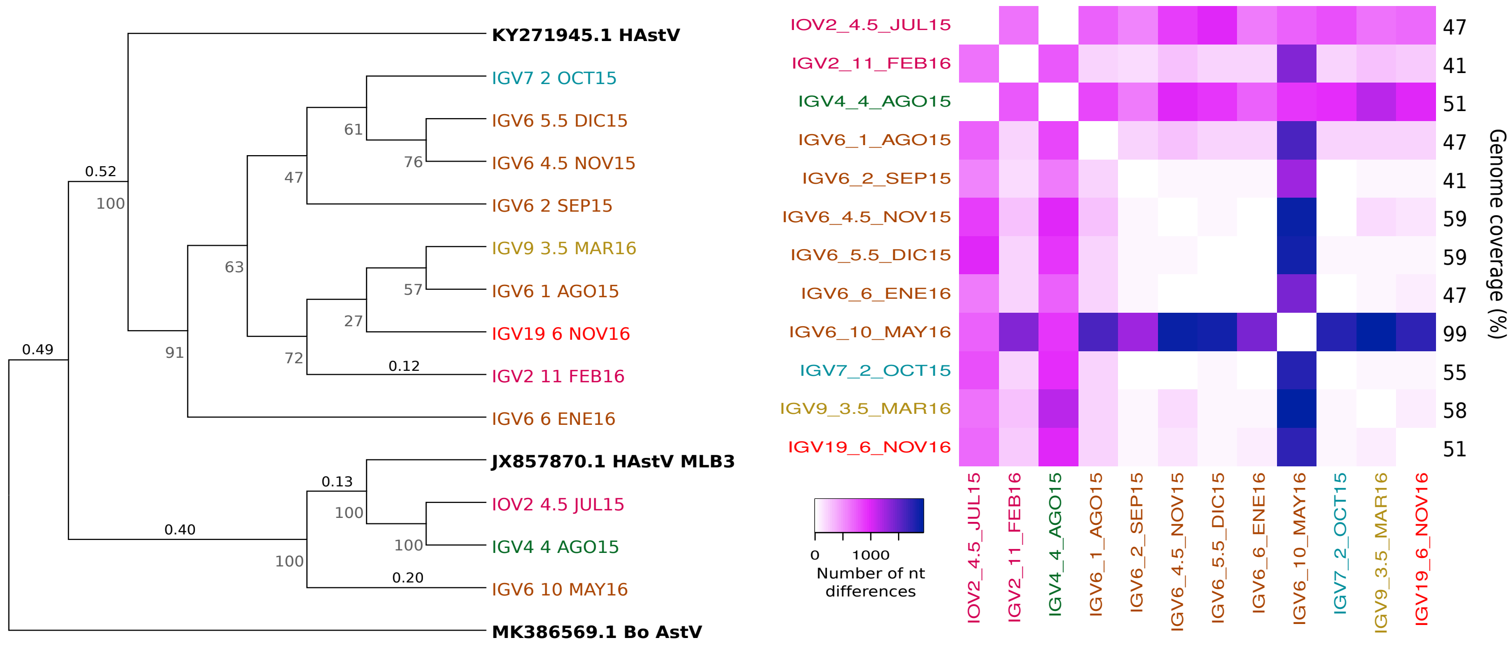


Fig. S10. Astrovirus genetic diversity. (a) Phylogenetic tree of complete genomes and partial sequences (contigs > 500 nt) constructed with TIM2+F+I model. (b) Heatmap showing pairwise nucleotide differences of viral sequences with at least 40% of the genome coverage. Reference and rooted strains are in bold. Each children has an assigned color and names follow the next code: "IOV19_5 SEP16" indicates Infant Oropharynx Virus collected from child 19 at 5 months of age, on September 2016; "IGV11_1.5 DEC15" indicates Infant Gastrointestinal Virus collected from child 11 at 1.5 months of age, on December 2015.

# Parechovirus

**a** **b**

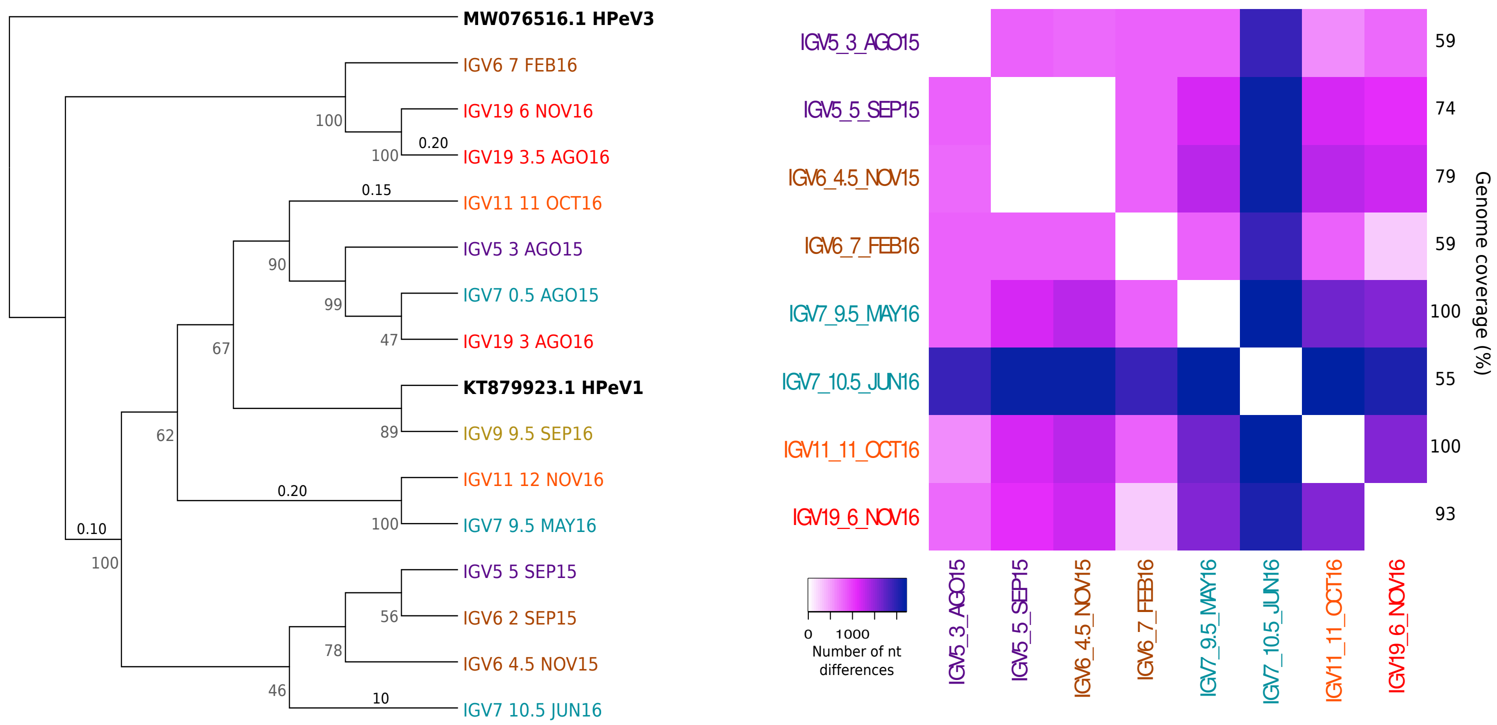


Fig. S11. Parechovirus genetic diversity. (a) Phylogenetic tree of complete genomes and partial sequences (contigs > 500 nt) constructed with TIM2+F+R4 model. (b) Heatmap showing pairwise nucleotide differences of viral sequences with at least 40% of the genome coverage. Reference and rooted strains are in bold. Each children has an assigned color and names follow the next code: "IOV19_5 SEP16" indicates Infant Oropharynx Virus collected from child 19 at 5 months of age, on September 2016; "IGV11_1.5 DEC15" indicates Infant Gastrointestinal Virus collected from child 11 at 1.5 months of age, on December 2015.

# Anellovirus

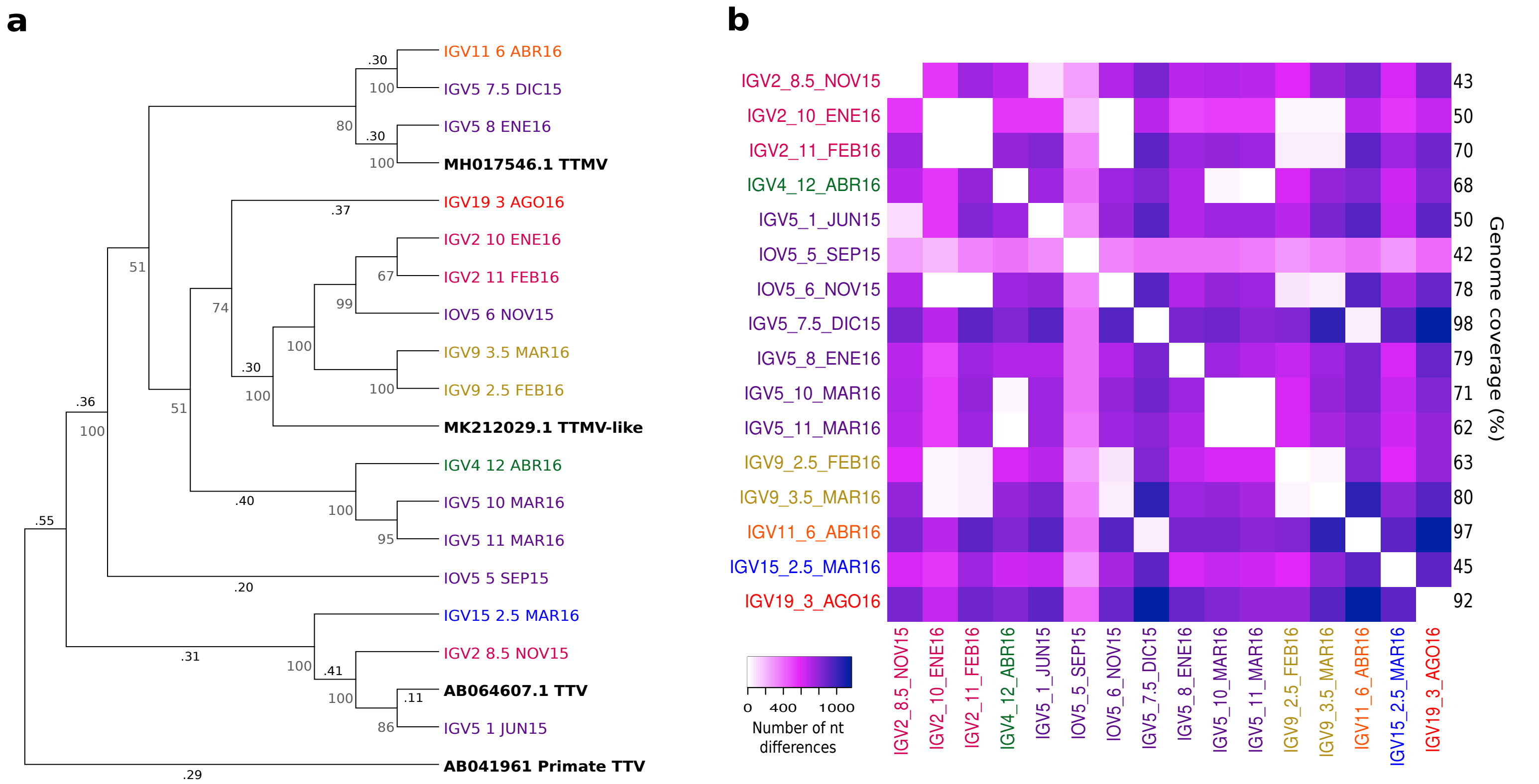


Fig. S12. Anellovirus genetic diversity. (a) Phylogenetic tree of complete genomes and partial sequences (contigs > 500 nt) constructed with TIM2+F+R2 model. (b) Heatmap showing pairwise nucleotide differences of viral sequences with at least 40% of the genome coverage. Reference and rooted strains are in bold. Each children has an assigned color and names follow the next code: "IOV19_5 SEP16" indicates Infant Oropharynx Virus collected from child 19 at 5 months of age, on September 2016; "IGV11_1.5 DEC15" indicates Infant Gastrointestinal Virus collected from child 11 at 1.5 months of age, on December 2015.

# Papillomavirus

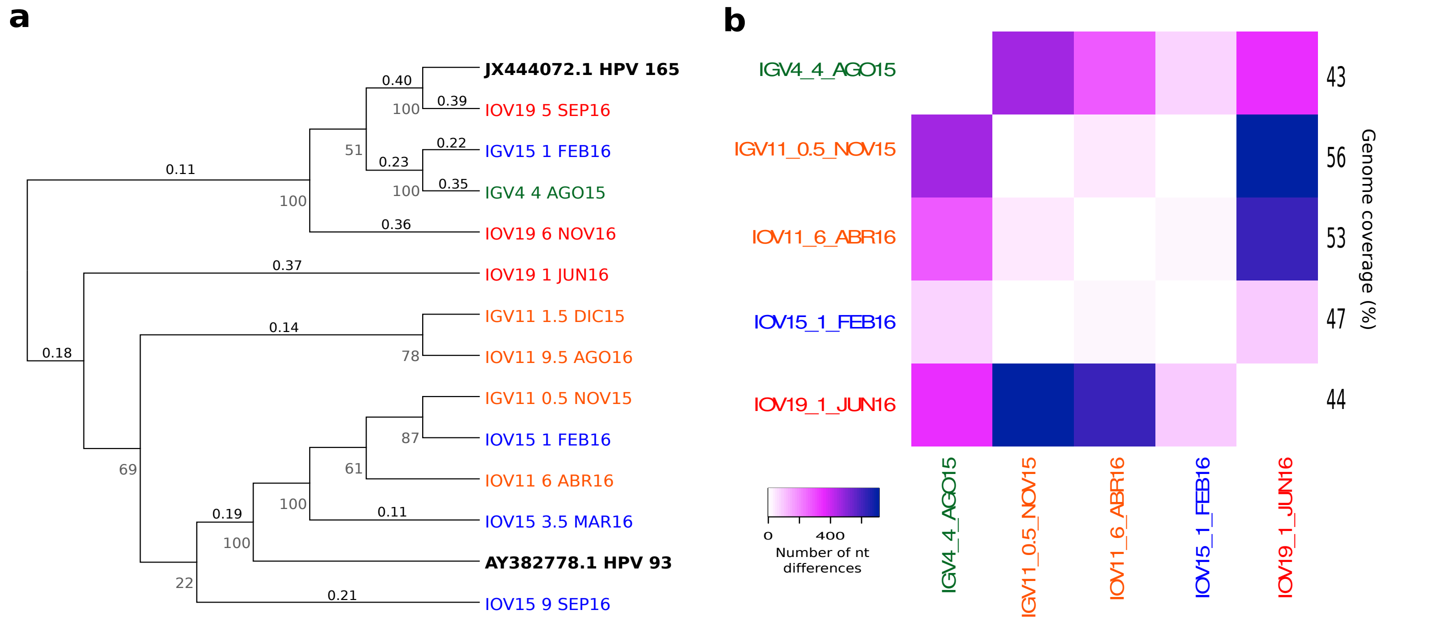


Fig. S13. Human papillomavirus genetic diversity. (a) Phylogenetic tree of complete genomes and partial sequences (contigs > 500 nt) constructed with TVM2+F+I model. (b) Heatmap showing pairwise nucleotide differences of viral sequences with at least 40% of the genome coverage. Reference and rooted strains are in bold. Each children has an assigned color and names follow the next code: "IOV19_5 SEP16" indicates Infant Oropharynx Virus collected from child 19 at 5 months of age, on September 2016; "IGV11_1.5 DEC15" indicates Infant Gastrointestinal Virus collected from child 11 at 1.5 months of age, on December 2015.

# Rhinovirus

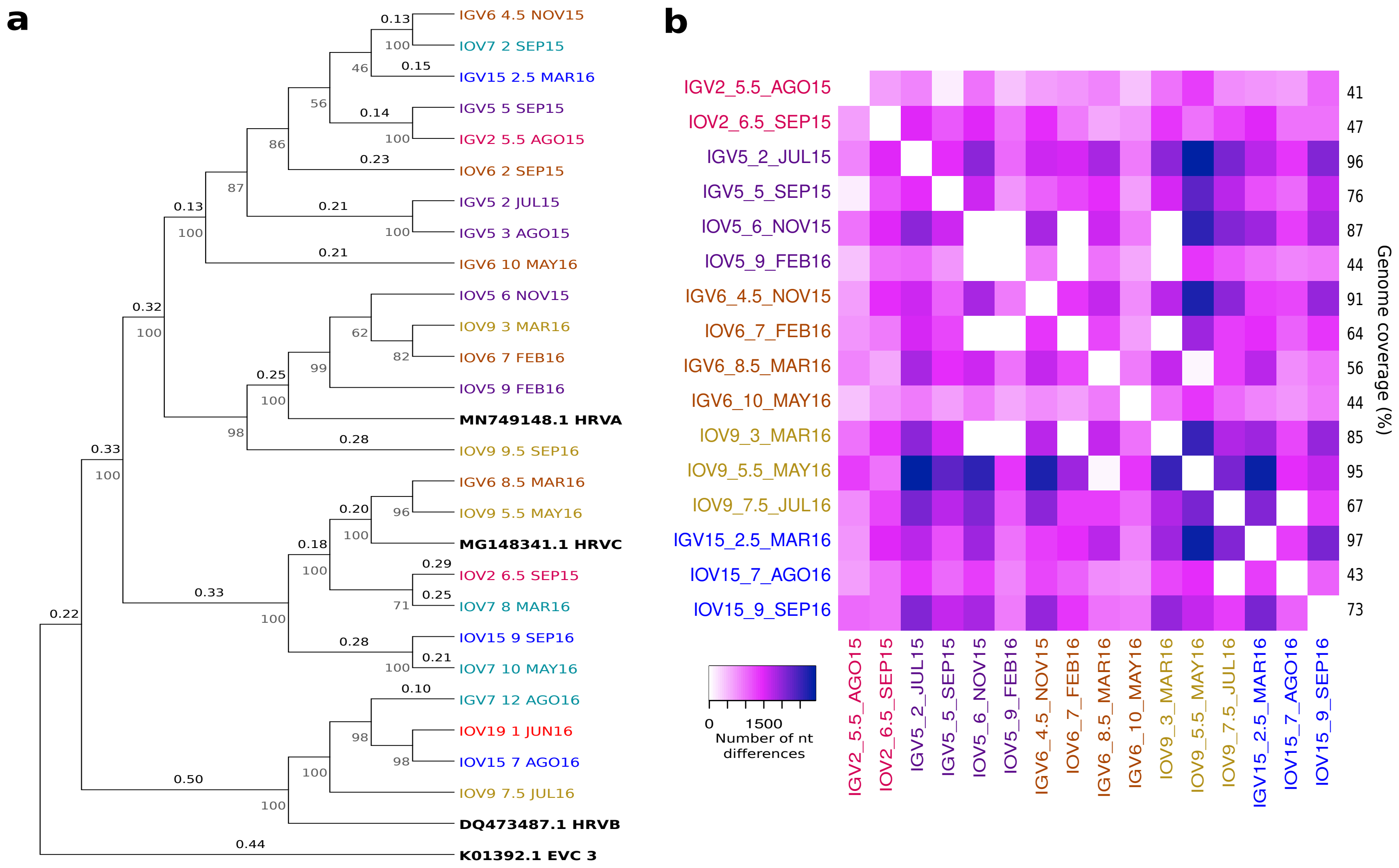


Fig. S14. Human rhinovirus genetic diversity. (a) Phylogenetic tree of complete genomes and partial sequences (contigs > 500 nt) constructed with GTR+F+I+G4 model. (b) Heatmap showing pairwise nucleotide differences of viral sequences with at least 40% of the genome coverage. Reference and rooted strains are in bold. Each children has an assigned color and names follow the next code: "IOV19_5 SEP16" indicates Infant Oropharynx Virus collected from child 19 at 5 months of age, on September 2016; "IGV11_1.5 DEC15" indicates Infant Gastrointestinal Virus collected from child 11 at 1.5 months of age, on December 2015.

# Bocavirus

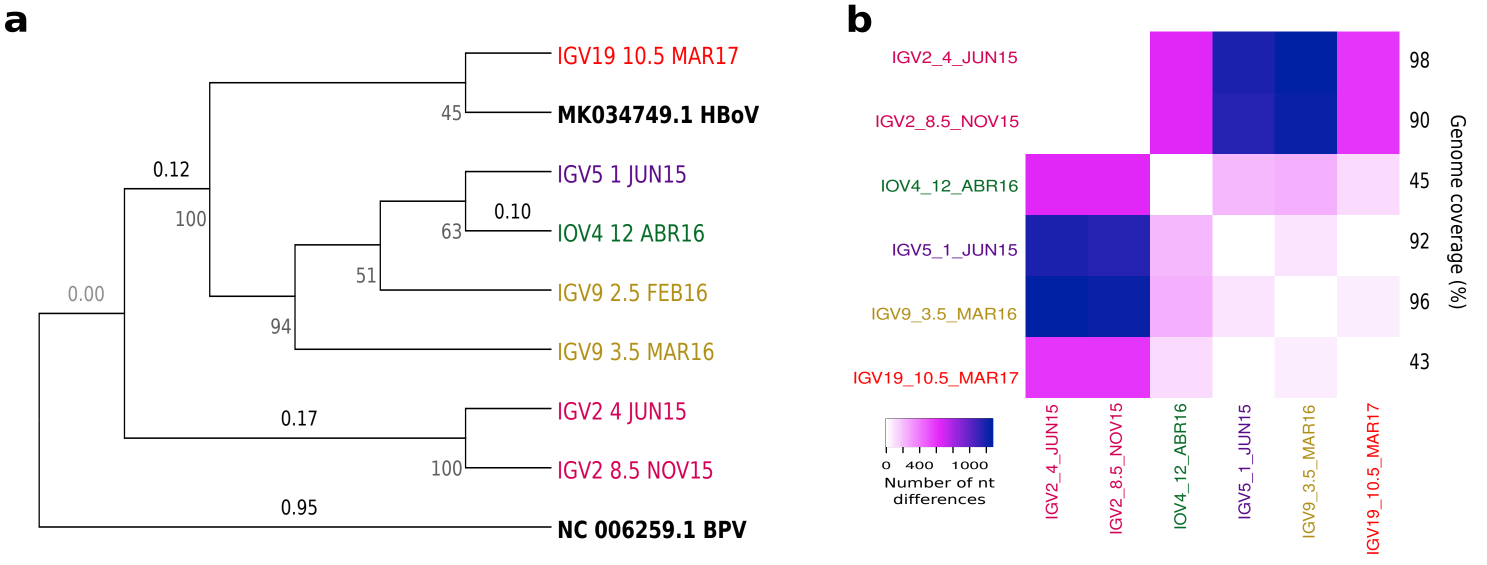


Fig. S15. Human bocavirus genetic diversity. (a) Phylogenetic tree of complete genomes and partial sequences (contigs > 500 nt) constructed with HKY+F model. (b) Heatmap showing pairwise nucleotide differences of viral sequences with at least 40% of the genome coverage. Reference and rooted strains are in bold. Each children has an assigned color and names follow the next code: "IOV19_5 SEP16" indicates Infant Oropharynx Virus collected from child 19 at 5 months of age, on September 2016; "IGV11_1.5 DEC15" indicates Infant Gastrointestinal Virus collected from child 11 at 1.5 months of age, on December 2015.

# Tropical soda apple mosaic virus

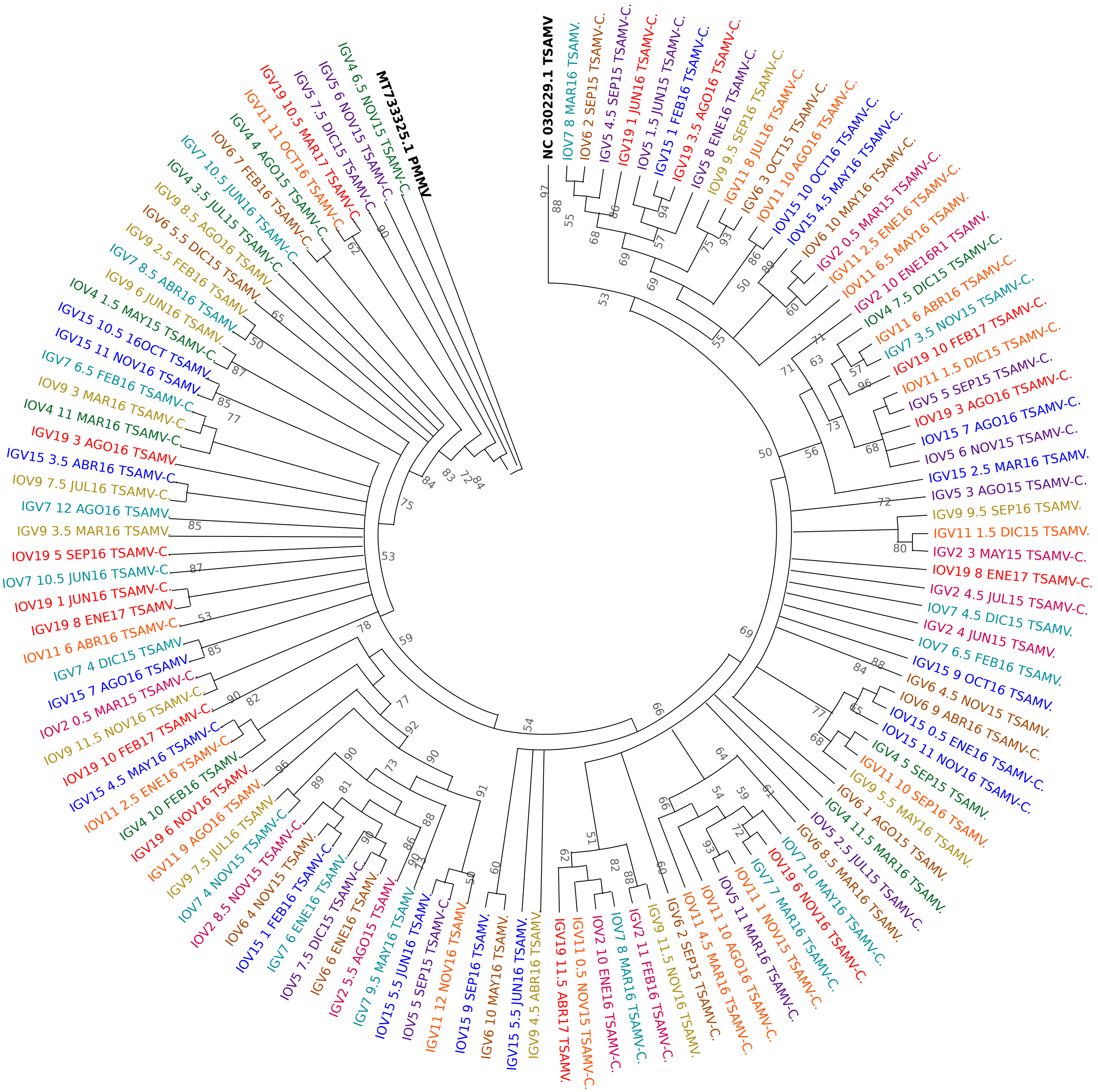


Fig. S16. Tropical soda apple mosaic virus genetic diversity. Phylogenetic tree of complete genomes and partial sequences (contigs > 500 nt) constructed with GTR+F+R6 model. Reference and rooted strains are in bold. Each children has an assigned color and names follow the next code: "IOV19_5 SEP16" indicates Infant Oropharynx Virus collected from child 19 at 5 months of age, on September 2016; "IGV11_1.5 DEC15" indicates Infant Gastrointestinal Virus collected from child 11 at 1.5 months of age, on December 2015.

# Tropical soda apple mosaic virus

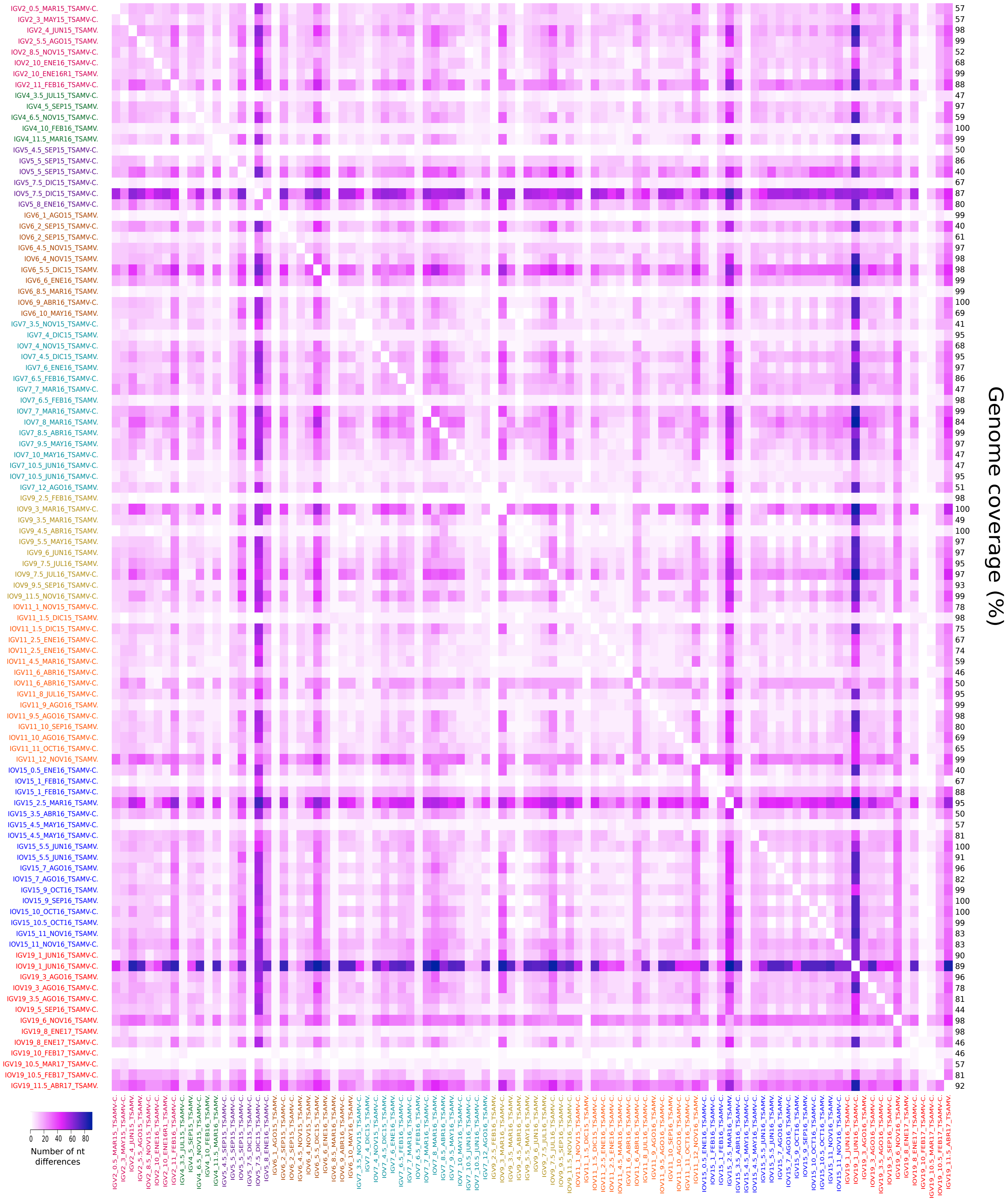


Fig. S17. Tropical soda apple mosaic virus nucleotide differences. Heatmap of pairwise nucleotide differences was constructed with viral sequences with at least 40% of the genome coverage. Each children has an assigned color and names follow the next code: "IOV19_5 SEP16" indicates Infant Oropharynx Virus collected from child 19 at 5 months of age, on September 2016; "IGV11_1.5 DEC15" indicates Infant Gastrointestinal Virus collected from child 11 at 1.5 months of age, on December 2015.

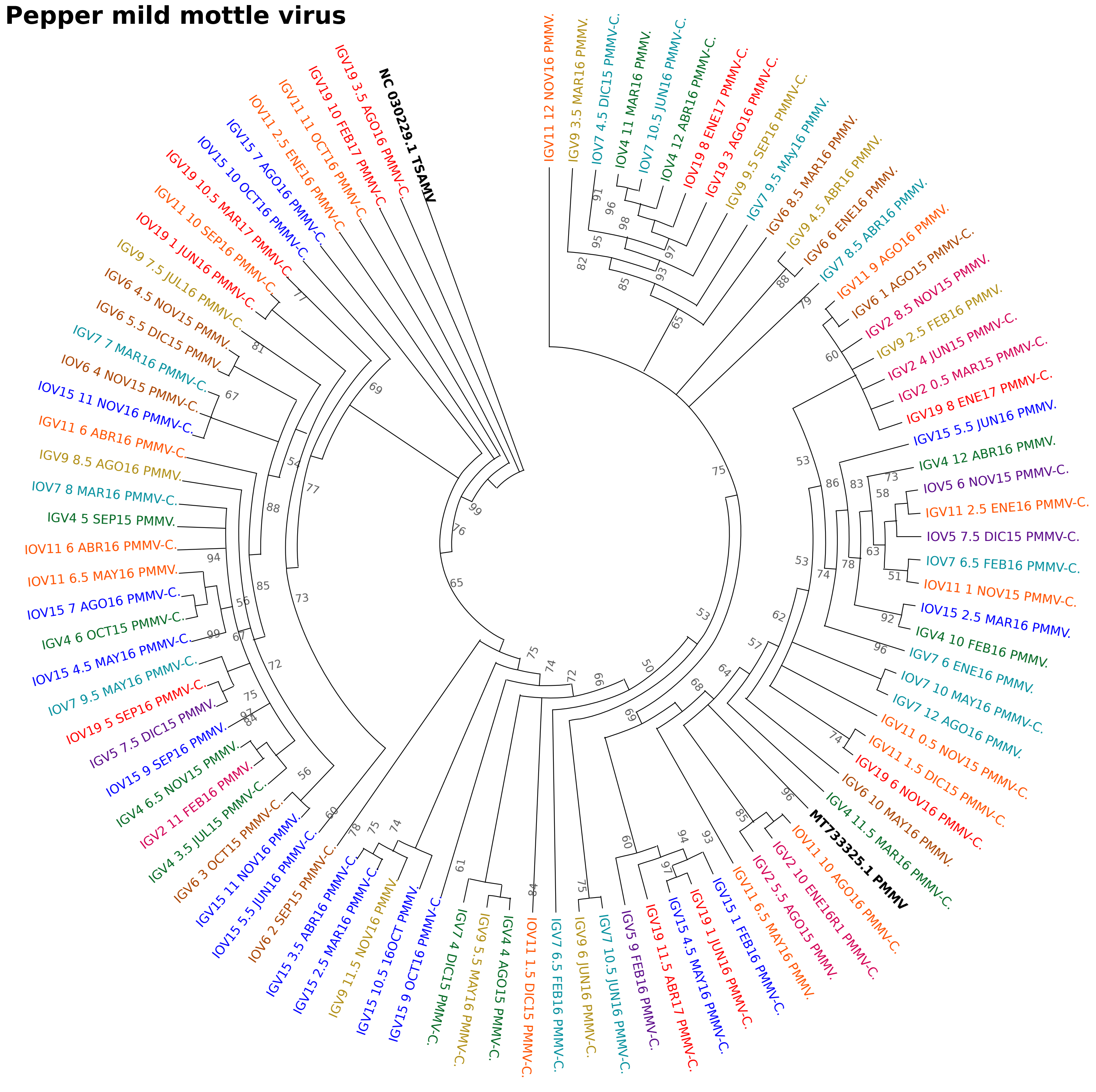


Fig. S18. Pepper mild mottle virus genetic diversity. Phylogenetic tree of complete genomes and partial sequences (contigs > 500 nt) constructed with GTR+F+R10 model. Reference and rooted strains are in bold. Each children has an assigned color and names follow the next code: "IOV19_5 SEP16" indicates Infant Oropharynx Virus collected from child 19 at 5 months of age, on September 2016; "IGV11_1.5 DEC15" indicates Infant Gastrointestinal Virus collected from child 11 at 1.5 months of age, on December 2015.

# Pepper mild mottle virus

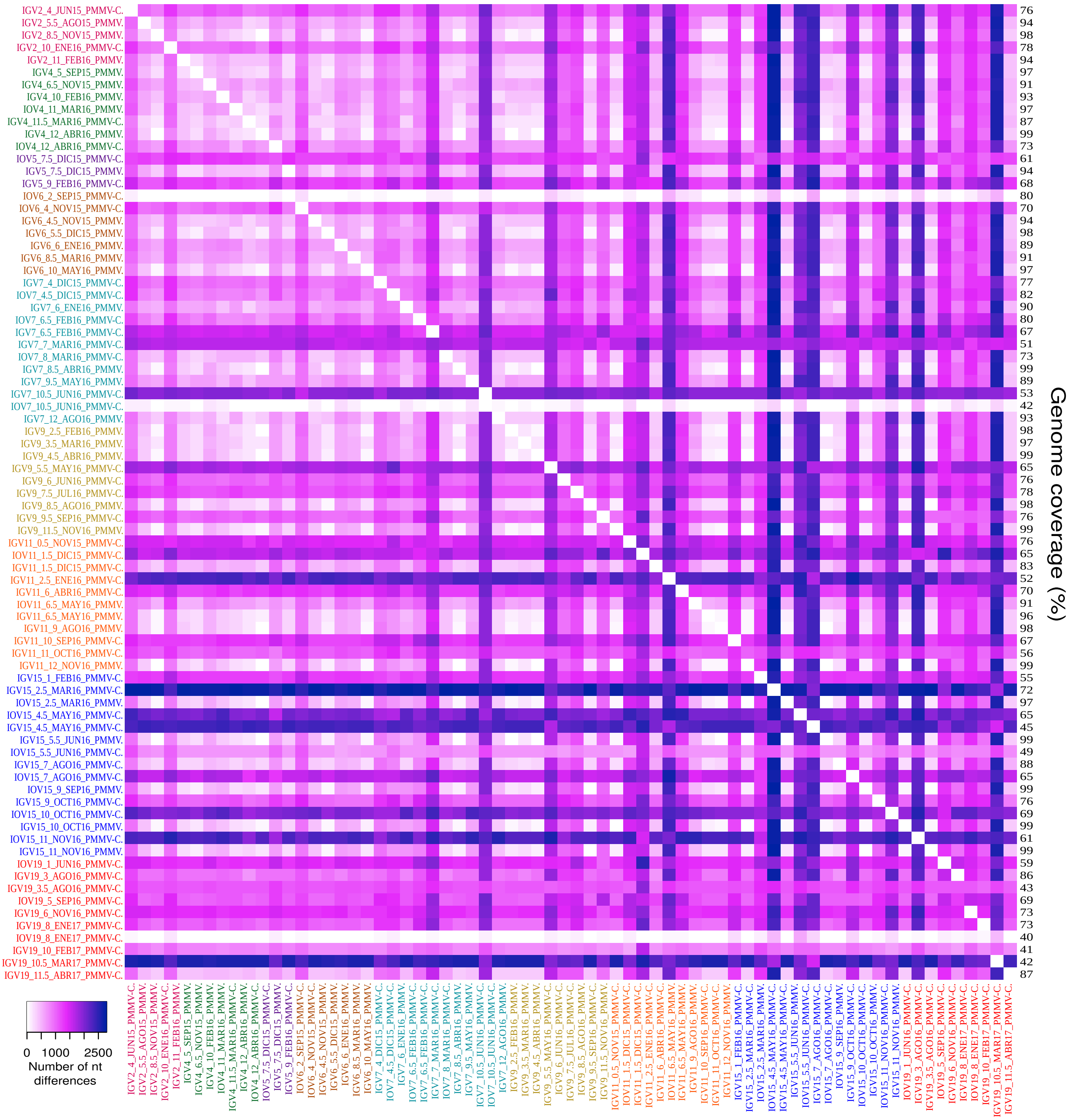


Fig. S19. Pepper mild mottle virus nucleotide differences. Heatmap of pairwise nucleotide differences was constructed with viral sequences with at least 40% of the genome coverage. Each children has an assigned color and names follow the next code: "IOV19_5 SEP16" indicates Infant Oropharynx Virus collected from child 19 at 5 months of age, on September 2016; "IGV11_1.5 DEC15" indicates Infant Gastrointestinal Virus collected from child 11 at 1.5 months of age, on December 2015.
